# A novel NF-κB regulator encoded by circPLCE1 inhibits colorectal carcinoma progression by promoting RPS3 ubiquitin-dependent degradation

**DOI:** 10.1186/s12943-021-01404-9

**Published:** 2021-08-19

**Authors:** Zhen-xing Liang, Hua-shan Liu, Li Xiong, Xin Yang, Feng-wei Wang, Zi-wei Zeng, Xiao-wen He, Xian-rui Wu, Ping Lan

**Affiliations:** 1 Department of Colorectal Surgery, The Sixth Affiliated Hospital, Sun Yat-Sen University, 26 Yuancun Erheng Rd, Guangzhou, Guangdong, 510655 China; 2 Guangdong Provincial Key Laboratory of Colorectal and Pelvic Floor Diseases, The Sixth Affiliated Hospital, Sun Yat-Sen University, Guangzhou, Guangdong China; 3 Bioland Laboratory, Guangzhou Regenerative Medicine and Health Guangdong Laboratory, Guangzhou, China; 4 Department of Endocrinology, The First Affiliated Hospital of Sun Yat-Sen University, Guangzhou, China; 5 State Key Laboratory of Oncology in South China, Sun Yat-Sen University Cancer Center, Guangzhou, Guangdong China

**Keywords:** NF-κB, Colorectal carcinoma, Circular RNA, circPLCE1

## Abstract

**Background:**

Constitutive activation of nuclear factor-κB (NF-κB) signaling plays a key role in the development and progression of colorectal carcinoma (CRC). However, the underlying mechanisms of excessive activation of NF-κB signaling remain largely unknown.

**Methods:**

We used high throughput RNA sequencing to identify differentially expressed circular RNAs (circRNAs) between normal human intestinal epithelial cell lines and CRC cell lines. The identification of protein encoded by circPLCE1 was performed using LC–MS. The function of novel protein was validated in vitro and in vivo by gain or loss of function assays. Mechanistic results were concluded by immunoprecipitation analyses.

**Results:**

A novel protein circPLCE1-411 encoded by circular RNA circPLCE1 was identified as a crucial player in the NF-κB activation of CRC. Mechanistically, circPLCE1-411 promoted the ubiquitin-dependent degradation of the critical NF-κB regulator RPS3 via directly binding the HSP90α/RPS3 complex to facilitate the dissociation of RPS3 from the complex, thereby reducing NF-κB nuclear translocation in CRC cells. Functionally, circPLCE1 inhibited tumor proliferation and metastasis in CRC cells, as well as patient-derived xenograft and orthotopic xenograft tumor models. Clinically, circPLCE1 was downregulated in CRC tissues and correlated with advanced clinical stages and poor survival.

**Conclusions:**

circPLCE1 presents an epigenetic mechanism which disrupts NF-κB nuclear translocation and serves as a novel and promising therapeutic target and prognostic marker.

**Supplementary Information:**

The online version contains supplementary material available at 10.1186/s12943-021-01404-9.

## Background

Nuclear factor-κB (NF-κB) is one of the most pleiotropic transcription factors [1–3]. Numerous studies have revealed that constitutive activation of NF-κB signaling pathway plays important roles in the development and progression of human cancers [4–6]. Moreover, excessive activation of NF-κB in tumor was demonstrated to be associated with advanced tumor stage as well as poor overall survival [7–9]. However, the mechanisms of constitutive activation of the NF-κB signaling pathway remain incompletely understood.

Colorectal carcinoma (CRC) represents a frequently encountered fatal disease entity worldwide [10]. Due to limitations in therapeutic methods for the treatment of distant metastasis, it still accounts for ~ 90% of CRC-related death [11, 12]. Therefore, the need to explore the factors which drive tumor initiation and metastasis is urgent so as to provide better therapeutic options and improve the oncological outcomes of CRC patients. Constitutive activation of NF-κB was shown to be correlated with advanced tumor stages and poor survival of CRC patients [7, 13]. In CRC development, NF-κB has been reported to play vital roles from early adenoma to invasive cancer and metastasis [6]. Aberrant activation of NF-κB increased Wnt signaling pathway and induced dedifferentiation of non-stem cells that acquired tumor-initiating capacity to initiate tumourigenesis [14]. Moreover, NF-κB promoted CRC progression via enhancing epithelial-mesenchymal transition, facilitating autonomous growth signaling and remodeling tumor microenvironment [15–17]. However, the underlying mechanisms of excessive activation of NF-κB signaling pathway remain largely unknown in CRC.

Circular RNAs (circRNAs) are a group of endogenous non-coding RNAs, circularized by a back-splicing reaction [18, 19]. Given their stability and evolutionary conservation, circRNAs may serve as important regulators of various cellular biological and pathological processes [20]. Up to date, most studies on circRNAs focused on their functions as microRNA/protein sponges [21, 22]. It was assumed that circRNAs were untranslatable because of the absence of open reading frames (ORFs). However, recent studies showed that circRNAs could indeed encode proteins [23, 24]. The study by Zhang indicated that circ-E-Cad was able to encode C-E-Cad which promoted glioblastoma tumorigenicity through activation of EGFR-STAT3 signaling pathway [25]. In colon cancer, circRNAs, such as circPPP1R12A and circFNDC3B have also been shown to regulate tumor progression by encoding novel proteins [24, 26]. With regard to the relationship between circRNAs and NF-κB signaling pathway in CRC, Chen and colleagues reported that circGLIS2 maintained the abnormal activation state of the NF-κB signaling pathway via the miR-671 sponge mechanism [27]. However, it remains unclear whether there are protein-coding circRNAs involved in the regulation of the NF-κB signaling.

In this study, we found that the circRNA circPLCE1 was downregulated in CRC tissues and was associated with clinical stages and survival. Furthermore, circPLCE1 promoted CRC proliferation and metastasis both in vitro and in vivo. Mechanistically, circPLCE1 encoded a novel protein, circPLCE1-411, to promote the ubiquitin-dependent degradation of the critical NF-κB regulator RPS3 via directly binding the HSP90α/RPS3 complex, thereby promoting the dissociation of RPS3 from the complex and reducing NF-κB nuclear translocation.

## Methods

Detailed procedures are provided in Supplemental Methods.

### Cell lines and cell cultures

The human CRC cell lines HCT8 (p53-wt, KRAS-wt, APC- mut), DLD1 (p53-mut, KRAS-mut, APC-mut), normal human intestinal epithelial cell lines (HIEC-6 and NCM460) and human embryonic kidney 293 T cells were purchased from American Type Culture Collection (ATCC). All of the cells were cultured at 37 °C in Dulbecco’s Modified Eagle Medium (DMEM; Gibco, Thermo Fisher Scientific, St Peters, MO, USA) supplemented with 10% fetal bovine serum (FBS; Gibco, Thermo Fisher Scientific, St Peters, MO, USA) in a 5% CO2 atmosphere.

### RNA sequencing

Total RNA was extracted from normal human intestinal epithelial cell lines (HIEC-6 and NCM460) and CRC cell lines (HCT8, HCT116 and DLD1). The RNA purity was analyzed on a Bioanalyzer 2200 instrument (Aligent). Then the RNA was treated with RiboMinus Eukaryote Kit (Qiagen, Valencia, CA) to remove ribosomal RNA and a cDNA library was constructed. Finally deep sequencing was performed with an Illumina HiSeq 3000 (Illumina, San Diego, CA). The clean reads were aligned to the reference genome (GRCH37.p13 NCBI). Unmapped reads were collected to identify the circRNAs. Reads that mapped to the circRNA junction (with an overhang of at least 6 nt) were counted for each candidate.

### Patients and samples

Eighty-five paired CRC samples and normal adjacent tissues were used to analyze circPLCE1 RNA levels. None of the patients received chemotherapy or radiotherapy before surgery. All the samples were collected from the Sixth Affiliated Hospital of Sun Yat-sen University. All samples were stored at -80 °C refrigerator until further use.

### Animal experiments

For the construction of orthotopic xenograft CRC mouse model, 2 × 10^6^ HCT8 cells transfected with empty vector, circPLCE1 vector, circPLCE1-ATGmut vector or circPLCE1-411 vector were injected into the wall of the cecum in 6-week-old NOD-SCID mice. Each group consisted of 5 mice. After 8 weeks, all the mice were sacrificed. Intestines, livers were harvested to assess the tumor burden. Cryosections of the harvested organs were stained using H&E for histological assessment. RNA from the rest of organs was extracted for qRT-PCR analysis of human hypoxanthine phosphoribosyltransferase (HPRT) mRNA expression.

For the construction of CRC PDX model, fresh tumor tissues were obtained from two CRC patients and implanted into NCG mice. When the tumor size reached 1.5 cm^3^, the tumors were divided into equal volume ~ 2 mm^3^ and were subcutaneously implanted into 4–5 weeks old male NOD-SCID mice. When the tumor size reached about 100 mm^3^, all mice were randomized into four groups (n = 5 per group): empty group, circPLCE1 group, circPLCE1-ATGmut group and circPLCE1-411 group and treated with intratumor injection of appropriate lentivirus. All mice were sacrificed 4 weeks later and subcutaneous tumors were subjected to H&E and IHC analysis.

### Statistical analysis

GraphPad Prism Software (GraphPad Software, La Jolla, CA, USA) was used to perform statistical analysis. Two-tailed Student’s *t* test and one-way ANOVA analysis were performed for statistical comparisons. All statistics analysis data are expressed as mean ± standard error of the mean. A *p* value < 0.05 was considered statistically significant.

## Results

### Characterization and clinical signification of circPLCE1 in CRC

**Fig. 1:**
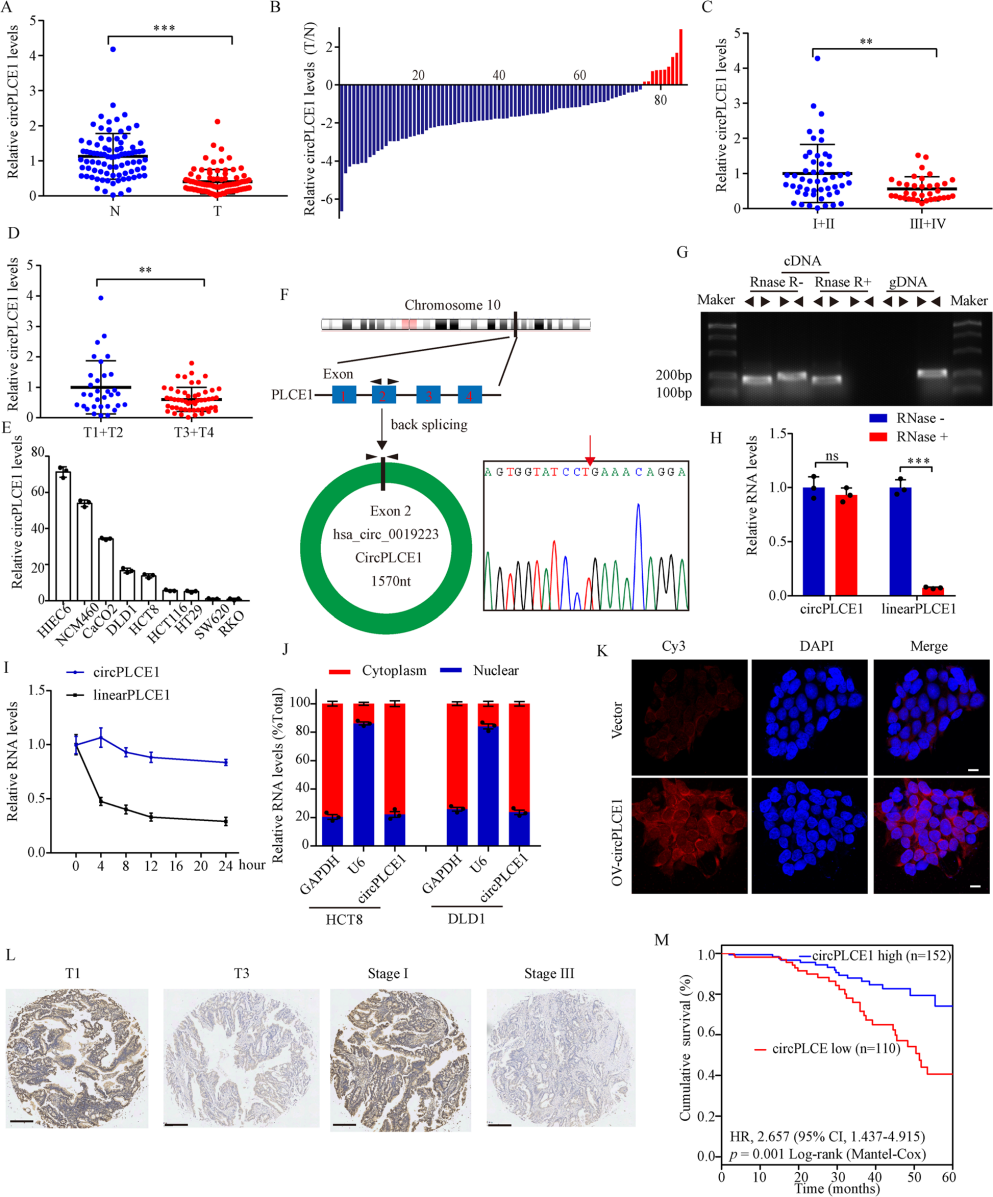
Characterization and clinical signification of circPLCE1. **A** qRT-PCR analysis of circPLCE1 expression in 85 paired CRC samples and normal adjacent tissues. **B** Fold change of circPLCE1 expression in 85 paired CRC tissues. **C** Comparison of circPLCE1 expression between patients with clinical stage III–IV (n = 35) and those with clinical stage I–II (n = 50), detected by qRT-PCR. **D** Comparison of circPLCE1 expression between patients with T stage 3–4 (n = 53) and those with T stage 1–2 (n = 32), detected by qRT-PCR. **E** qRT-PCR analysis of circPLCE1 expression in normal human intestinal epithelial and CRC cell lines, n = 3. **F** Genomic loci of the PLCE1 gene and circPLCE1. Red arrow indicates the back-splicing of PLCE1 exon 2 confirmed by Sanger sequencing. **G** RT-PCR analysis of the existence of circPLCE1 with the divergent primers and convergent primers in complementary DNA (cDNA) and genomic DNA (gDNA). **H** qRT-PCR analysis of circPLCE1 and PLCE1 linear mRNA treated with or without RNase R, n = 3. **I** qRT-PCR analysis of the abundance of circPLCE1 and PLCE1 linear mRNA in HCT8 cells treated with actinomycin D at the indicated time points, n = 3. **J** qRT-PCR analysis of circPLCE1 location in the nucleus or cytoplasm in HCT8 cells. GADPH served as a marker of cytoplasmic location, whileU6 served as a marker of nuclear location, n = 3. **K** Representative images for FISH circPLCE1 staining in HCT8 cells. Scale bar = 10 μm. **L** Representative images of ISH circPLCE1 expression in the paraffin-embedded CRC tissues of T stage T1 and T3 and clinical stage I and III. Scale bar = 250 μm. **M** Kaplan–Meier curves for survival of CRC patients with low *vs.* high expression of circPLCE1. N, normal adjacent tissues tissues; T, tumor tissues. Values are represented as mean ± SD. **p < 0.01, ***p < 0.001, ns (no significance), by 2-tailed Student’s *t* test

To identify the differentially expressed circRNAs in CRC, we performed total RNA sequencing using both normal human intestinal epithelial cell lines (HIEC-6 and NCM460) and CRC cell lines (HCT8, HCT116 and DLD1). We identified 14 upregulated and 17 downregulated circRNAs with fold changes > 2 or < 0.5, *p* < 0.05 and transcript abundance > 0, as shown in Figure S1A. Six circRNAs with good consistency in expression levels between normal human intestinal epithelial cell lines and CRC cell lines were chosen. The expression levels of the 6 circRNAs were then detected in 24 paired tissues by quantitative real-time PCR (qRT-PCR), which showed that only circPLCE1 (hsa_circ_0019223, named as circPLCE1) was differently expressed between CRC samples and normal adjacent tissues (Figure S1B). We further analyzed circPLCE1 expression in 85 paired CRC samples, which confirmed that circPLCE1 was downregulated in 88.2% (75/85) of CRC patients (Fig. 1A and B). Further analyses revealed that circPLCE1 expression was lower in patients with advanced clinical stages or T stages (Fig. 1C and D). Moreover, the difference in the expression levels of circPLCE1 between normal human intestinal epithelial cell lines and CRC cell lines was analyzed using qRT-PCR, which arrived at the similar results (Fig. 1E).

circPLCE1 was formed by the back-splicing of exon 2 of the phospholipase C epsilon-1 gene (PLCE1) with 1570nt. The back-splicing junction of circPLCE1 was confirmed by Sanger sequencing (Fig. 1F). Divergent primers and convergent primers were used to detect the circular transcripts and linear transcripts of PLCE1 in both complementary DNA (cDNA) and genomic DNA (gDNA). The results indicated that circular transcripts of PLCE1 could only be amplified in cDNA by the divergent primers (Fig. 1G). Moreover, circPLCE1 was observed to resist RNase R digestion (Fig. 1G and H). Half-life assays further showed that circPLCE1 was much more stable than circPLCE1 linear mRNA (Fig. 1I). Additionally, localization of circPLCE1 was examined by nuclear mass separation assays and fluorescence in situ hybridization (FISH) assays, which demonstrated the enrichment of circPLCE1 in the cytoplasm of CRC cells (Fig. 1J and K).

We next analyzed circPLCE1 expression in a large cohort of CRC patients by in situ hybridization (ISH; n = 262, Table S1). The results suggested that circPLCE1 expression was lower in patients with advanced clinical stages or T stages (Fig. 1L). Kaplan–Meier curves showed that lower circPLCE1 expression was associated with poorer survival in CRC patients (Fig. 1M). These data validated the circularity and clinical significance of circPLCE1.

### circPLCE1 inhibits CRC cell proliferation and migration in vitro

**Fig. 2:**
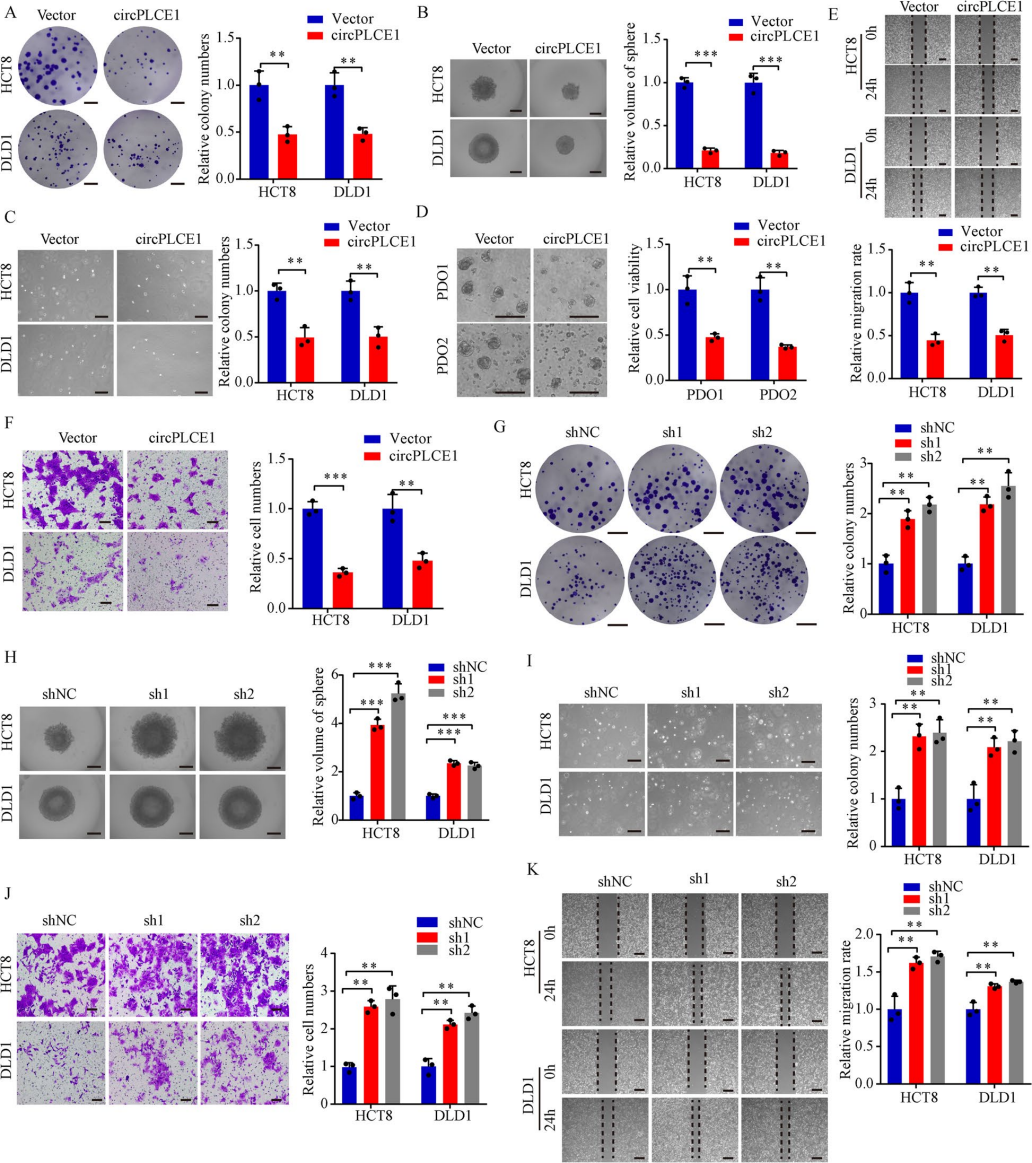
circPLCE1 inhibits CRC cell proliferation and metastasis in vitro. **A** Colony formation assays of circPLCE1 transfected HCT8 and DLD1 cells, n = 3. **B** Sphere formation assays of circPLCE1 transfected HCT8 and DLD1 cells, n = 3. Scale bar = 500 μm. **C** Anchorage-independent growth of circPLCE1 transfected HCT8 and DLD1 cells, n = 3. Scale bar = 200 μm. **D** Patient-derived organoids (PDOs) growth with circPLCE1 transfection, n = 3. Scale bar = 200 μm. **E** migration assays of circPLCE1 transfected HCT8 and DLD1 cells, n = 3. Scale bar = 100 μm. **F** wound-healing assays of circPLCE1 transfected HCT8 and DLD1 cells, n = 3. Scale bar = 100 μm. **G** Colony formation assays of circPLCE1 knockdown HCT8 and DLD1 cells, n = 3. **H** Sphere formation assays of circPLCE1 knockdown HCT8 and DLD1 cells, n = 3. Scale bar = 500 μm. **I** Anchorage-independent growth of circPLCE1 knockdown HCT8 and DLD1 cells, n = 3. Scale bar = 200 μm. **J** migration assays of circPLCE1 knockdown HCT8 and DLD1 cells, n = 3. Scale bar = 100 μm. K wound-healing assays of circPLCE1 knockdown HCT8 and DLD1 cells, n = 3. Scale bar = 100 μm. Values are represented as mean ± SD. **p < 0.01, ***p < 0.001, by 2-tailed Student’s *t* test (**A-F**) and one-way ANOVA (**G-K**)

To elucidate the functions of circPLCE1 in CRC, HCT8 and DLD1 CRC cell lines were selected to conduct cell experiments because of the moderate expression of circPLCE1 (Fig. 1E). We constructed CRC cell lines with stable overexpression of circPLCE1 (Figure S2A). circPLCE1 overexpression inhibited CRC cell colony formation, sphere formation and anchorage-independent growth (Fig. 2A-C). Moreover, we overexpressed circPLCE1 in two patient-derived organoids (PDOs) and the results also indicated that circPLCE1 decreased the growth of PDOs (Fig. 2D). Also, overexpression of circPLCE1 inhibited cell migration and invasion (Fig. 2E and F). Next, to construct CRC cell lines with stable knockdown of circPLCE1, we designed two short hairpin RNAs (shRNAs) which specifically targeted the back-spliced junction site of circPLCE1 without influencing the expression of linear PLCE1 (Figure S2B and S2C). As expected, circPLCE1 knockdown significantly promoted CRC cell growth, migration and invasion (Fig. 2G-K). Collectively, these results suggested that circPLCE1 played an important role in the proliferation and migration of CRC cells.

### circPLCE1 encodes a novel 411 amino acid protein, circPLCE1-411

**Fig. 3:**
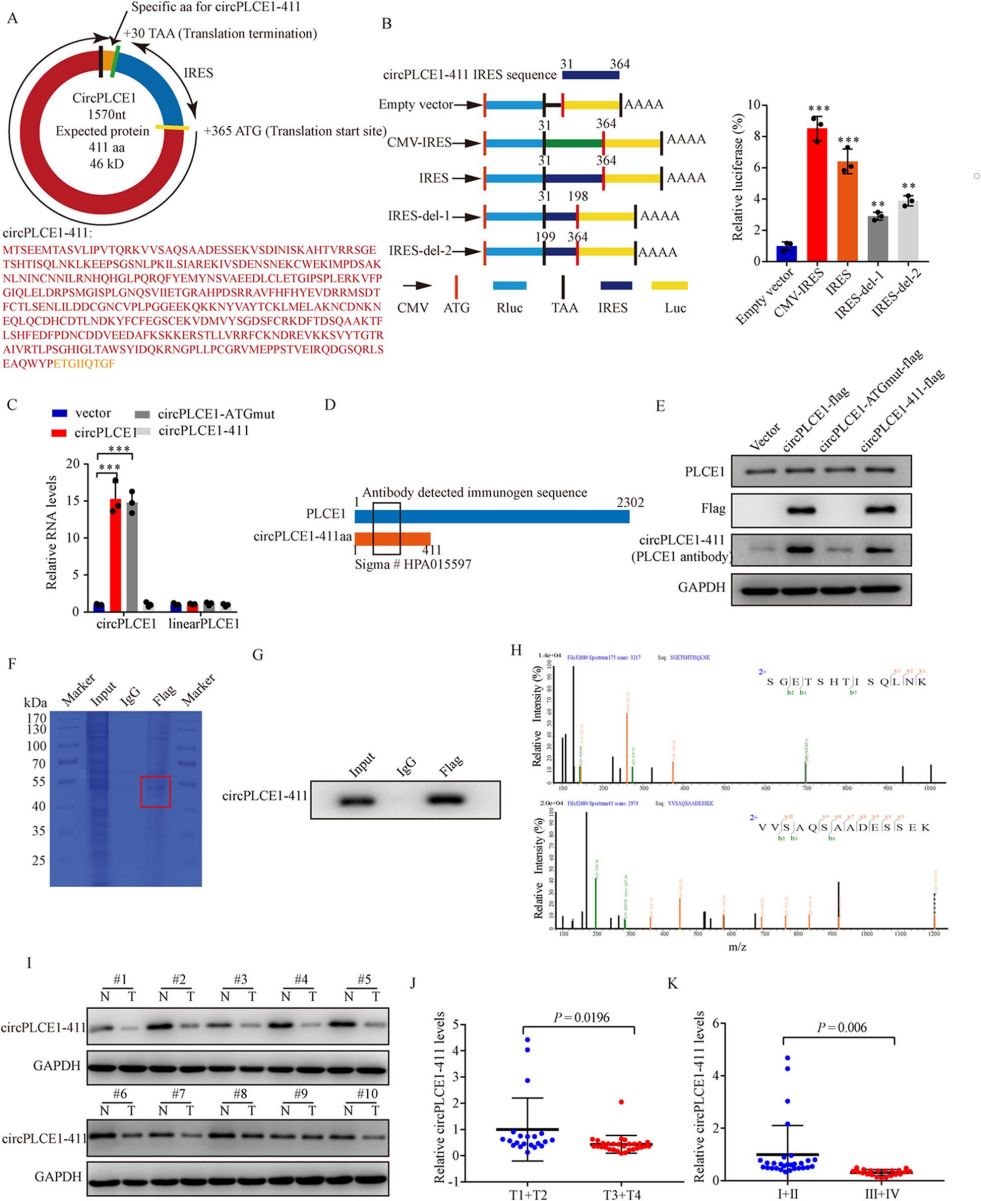
circPLCE1 encodes a 411 amino acid novel protein, circPLCE1-411. **A** Upper panel, the putative open reading frame (ORF) in circPLCE1. Lower panel, the sequences of the putative ORF encoded amino acid sequences are shown. **B** The putative IRES activity in circPLCE1 was tested. Left panel, IRES sequences in circPLCE1 or its different truncations were cloned between Rluc and Luc reporter genes with independent start and stop codons. Right panel, the relative luciferase activity of Luc/Rluc in the above vectors was tested, n = 3. **C** qRT-PCR analysis of circPLCE1 and PLCE1 linear mRNA expression in HCT8 cells transfected with empty vector, circPLCE1 vector, circPLCE1-ATGmut vector and circPLCE1-411 vector, n = 3. **D** Illustration of antibody detected immunogen sequence which could recognize both PLCE1 and circPLCE1-411 proteins. **E** Western blot analysis of PLCE1 and circPLCE1-411 protein levels in HCT8 cells transfected with empty vector, circPLCE1 vector, circPLCE1-ATGmut vector and circPLCE1-411 vector with indicated antibodies. **F** The lysates from immunoprecipitation assays were separated by SDS-PAGE. Protein bands near 50 kDa were excised manually and summited for identification by LC–MS/MS. **G** Western blot validation of circPLCE1-411 with anti-Flag and anti-PLCE1 antibody in immunoprecipitation products. **H** The identified circPLCE1-411 amino acids. **I** Western blot analysis circPLCE1-411 expression in paired CRC samples and normal adjacent tissues with indicated antibodies. **J** Comparison of circPLCE1-411 expression between patients with T stage 3–4 (n = 29) and those with T stage 1–2 (n = 21), detected by western blot. **K** Comparison of circPLCE1-411 expression between patients with clinical stage III–IV (n = 21) and those with clinical stage I–II (n = 29), detected by western blot. Values are represented as mean ± SD. **p < 0.01, ***p < 0.001, by 2-tailed Student’s *t* test (**J** and **K**) and one-way ANOVA (**B** and **C**)

To investigate the detailed mechanism of circPLCE1 in CRC, we first used the Coding Potential Assessment Tool to analyze the coding potential of circPLCE1which suggested the protein coding ability of circPLCE1 (coding probability > 0.999). We next analyzed the putative open reading frame (ORF) of circPLCE1 in ORF Finder (https://www.ncbi.nlm.nih.gov/orffinder/). There was a potential spanning junction ORF that encoded a 411 amino acid protein in circPLCE1 (referred as circPLCE1-411 hereafter, Fig. 3A). Moreover, conservation analysis implied that this ORF was translatable because it was highly conserved among different species (Table S2). The activity of the internal ribosome entry site (IRES) which drove the translation of the ORF was validated by dual-luciferase assays (Fig. 3B).

Next, we constructed four flag-labeled plasmids to confirm the existence of circPLCE1-411 (flag-labeled circPLCE1 sequence, flag-labeled circPLCE1 sequence with start codon mutant and flag-labeled circPLCE1-411 sequence). The expression of circPLCE1 and linear PLCE1 was measured by qRT-PCR (Fig. 3C). Besides, we found that the PLCE1 antibody (Sigma # HPA015597) shared the same immunogen sequence in circPLCE1-411 (Fig. 3D). Western blot assays indicated that circPLCE1-411 could be detected at approximate 50 kDa by PLCE1 and flag antibody without affecting the expression of PLCE1 (Fig. 3E). Moreover, qRT-PCR and western blot assays indicated that circPLCE1 or circPLCE1-411 did not affect the content of PLCE1 (Fig. 3C and E). To identify this protein, we performed immunoprecipitation assays with lysates from the cells transfected with flag-labeled circPLCE1 vector (Fig. 3F and G). The protein bands at approximate 50 kDa were excised and submitted for mass spectrometry (MS). Although the peptide sequences identified by MS could be found both in circPLCE1-411 and PLCE1 proteins (Fig. 3H, Figure S3A and S3B), the molecular weights of PLEC1 proteins were about 200 kDa, we therefore concluded that the peptides detected at approximately 50 kDa were derived from circPLCE1-411, a novel protein encoded by circPLCE1. These results proved that circPLCE1 encoded this novel protein. To explore the potential clinical implications of circPLCE1-411, we analyzed circPLCE1-411 expression in 50 paired CRC samples and normal adjacent tissues by western blot. The results showed that circPLCE1-411 was downregulated in the majority of CRC tissues (46/50) (Fig. 3I and Figure S3C). Moreover, circPLCE1-411 expression was inversely associated with the tumor clinical stages and T stages (Fig. 3J and K).

### circPLCE1 inhibits CRC cell proliferation and migration by encoding circPLCE1-411

**Fig. 4:**
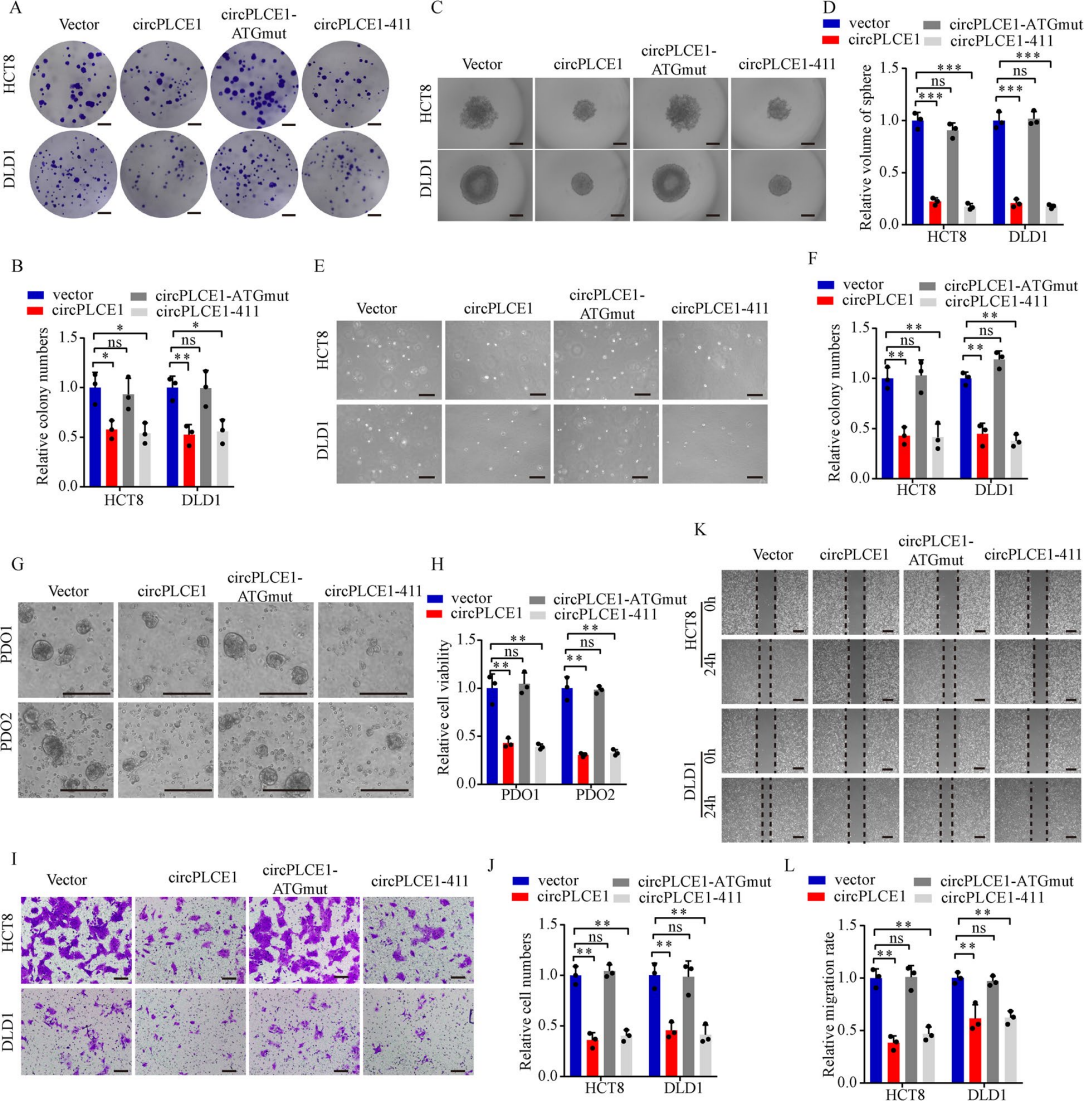
circPLCE1-411, not circPLCE1 itself, inhibits CRC cell progression. **A** and** B** Colony formation assays of HCT8 and DLD1 cells transfected with empty vector, circPLCE1 vector, circPLCE1-ATGmut vector and circPLCE1-411 vector, n = 3. **C** and** D** Sphere formation assays of HCT8 and DLD1 cells transfected with empty vector, circPLCE1 vector, circPLCE1-ATGmut vector and circPLCE1-411 vector, n = 3. Scale bar = 500 μm. **E** and **F** Anchorage-independent growth of HCT8 and DLD1 cells transfected with empty vector, circPLCE1 vector, circPLCE1-ATGmut vector and circPLCE1-411 vector, n = 3. Scale bar = 200 μm. **G** and **H** Patient-derived organoids (PDOs) growth with empty vector, circPLCE1 vector, circPLCE1-ATGmut vector and circPLCE1-411 vector transfection, n = 3. Scale bar = 200 μm. **I** and** J** migration assays of HCT8 and DLD1 cells transfected with empty vector, circPLCE1 vector, circPLCE1-ATGmut vector and circPLCE1-411 vector, n = 3. Scale bar = 100 μm. **K** and** L** wound-healing assays of HCT8 and DLD1 cells transfected with empty vector, circPLCE1 vector, circPLCE1-ATGmut vector and circPLCE1-411 vector, n = 3. Scale bar = 100 μm. Values are represented as mean ± SD. *p < 0.05, **p < 0.01, ***p < 0.001, ns (no significance), by one-way ANOVA

To explore the biological functions of circPLCE1-411, we performed cellular experiments using cells that were stably transfected with the plasmids as mentioned before (Fig. 3E). The results indicated that CRC cells transfected with circPLCE1 or circPLCE1-411 inhibited colony formation, sphere formation, anchorage-independent growth and PDOs growth. However, transfection with the circPLCE1 vector with the start codon mutant (circPLCE1-ATGmut) had no effect on CRC cell proliferation (Fig. 4A-H). Moreover, cell migration and wound healing assays also showed that transfection with the circPLCE1 or circPLCE1-411 vector inhibited CRC cell migration and invasion, whereas transfection with circPLCE1-ATGmut had no effect on CRC cell migration (Fig. 4I-L). These data suggested that circPLCE1 inhibits CRC cell proliferation and migration by encoding circPLCE1-411 instead of the circular RNA form of circPLCE1.

### circPLCE1-411 binds to the HSP90α/RPS3 complex to promote ubiquitin-dependent degradation of RPS3 to inhibit NF-κB signaling

PLCE1 is a member of the phospholipase C family, which are able to hydrolyze membrane phosphoinositol 4,5-bisphosphates (PIP2) to inositol-1,4,5-phosphate (IP3) and diacylglycerol (DAG) to generate a response to extracellular stimulation [28]. There are several functional domains in PLCE1, including a core domain which possesses the ability to hydrolyze PIP2, pleckstrin-homology PH and C2 domains, a GTP-exchanging domain at the C-terminus and two Ras-associating domains at the N-terminus [29, 30]. However, we found that circPLCE1-411 did not include any of the functional domains of PLCE1. Therefore, we hypothesized that circPLCE1-411 promoted CRC progression via binding to other intermediate molecules. In order to verify this hypothesis, we assessed circPLCE1-411-interacting proteins using immunoprecipitation and subsequent mass spectrometry analyses. Eight potential proteins with scores > 100 were identified (Table S3). Among those proteins, RPS3, an important regulator of NF-κB, was found to have the highest score. Interestingly, HSP90α, a partner of RPS3 and regulates its ubiquitin-dependent degradation [31, 32], was also identified. Therefore, we hypothesized that circPLCE1-411 might bind to HSP90α/RPS3 complex to regulate NF-κB signaling, thus inhibiting the progression of CRC.

**Fig. 5:**
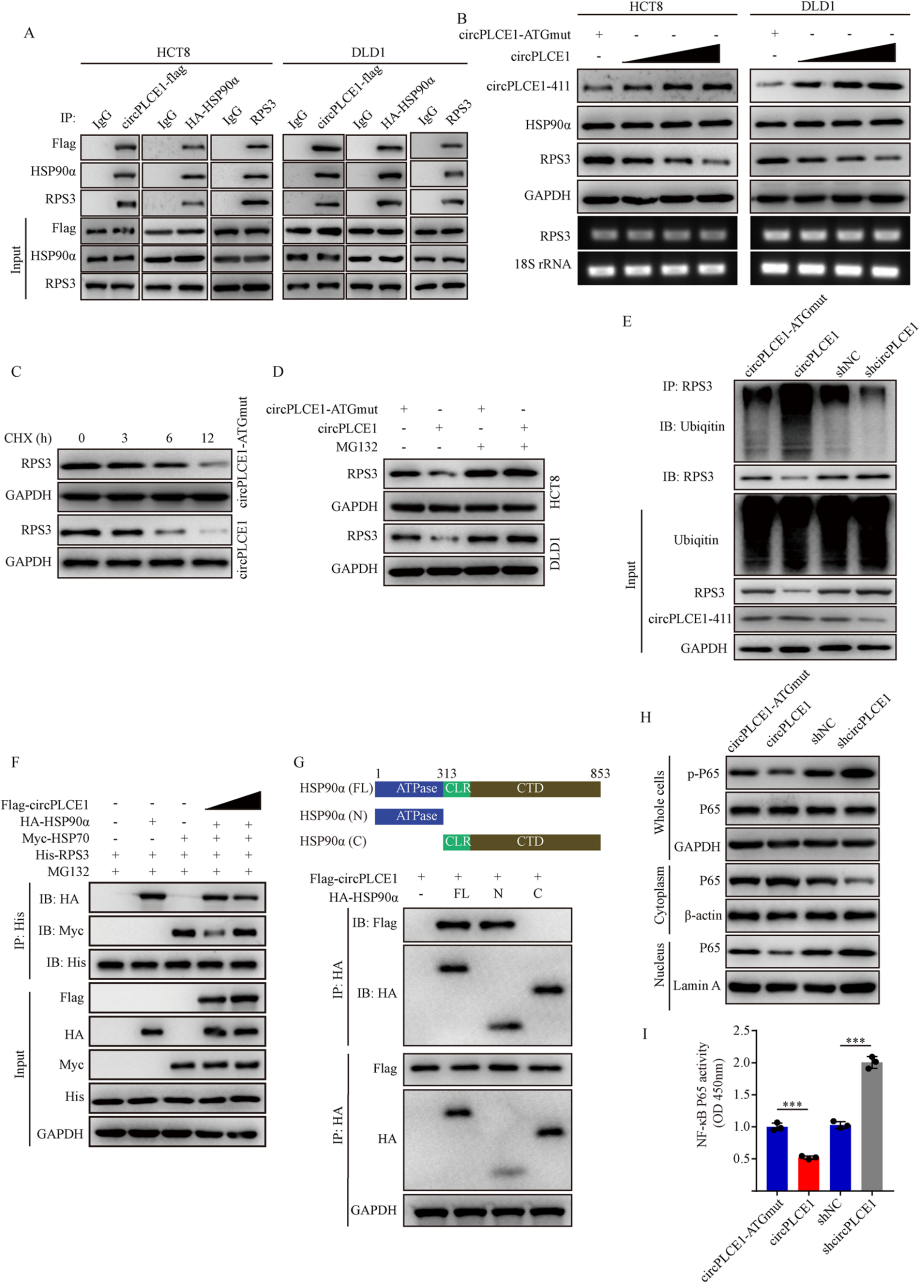
The mechanism of circPLCE1-411 inhibits NF-κB signaling. **A** Western blot analysis of immunoprecipitation using Flag, HA or RPS3 antibodies in HCT8 and DLD1 cells transfected with circPLCE1-flag vector or HA-HSP90α vector with indicated antibodies. **B** Western blot analysis of HCT8 and DLD1 cells transfected with circPLCE1-ATGmut or increasing circPLCE1 vector with indicated antibodies. Below, RT-PCR analysis of RPS3 mRNA; 18S rRNA serves as a loading control. **C** Western blot analysis of RPS3 protein levels in HCT8 cells transfected with circPLCE1-ATGmut or circPLCE1 with cycloheximide (CHX, 50 μg/ml) treatment at the indicated times. **D** Western blot analysis of RPS3 protein levels in HCT8 cells transfected with circPLCE1-ATGmut or circPLCE1 with MG132 (25 μM) for 12 h. **E** Western blot analysis of RPS3 ubiqitin levels after immunoprecipitation using RPS3 antibodies in HCT8 cells transfected with circPLCE1-ATGmut, circPLCE1, shNC or shcircPLCE1 vector. MG132 (25 μM) was added 6 h before harvest. **F** HEK293T cells were transfected with the indicated plasmids and MG132 (25 μM) was added simultaneously during transfection. Cell lysates were immunoprecipitated with anti-His antibody and then immunoblotted by the indicated antibodies. **G** Western blot analysis of immunoprecipitation using HA antibodies in HEK293T cells transfected with HA-HSP90α (FL), HA-HSP90α (N) and HA-HSP90α (C) with indicated antibodies. **H** Western blot analysis of p-P65 and P65 protein levels from whole-cell, nuclear, and cytoplasmic extracts in HCT8 cells transfected with the indicated plasmids. GAPDH, β-actin and Lamin A served as loading control. **I** Transcription factor binding assay of P65 in nuclear extracts obtained from HCT8 cells transfected with the indicated plasmids, n = 3. Values are represented as mean ± SD. ***p < 0.001, by 2-tailed Student’s *t* test (**I**)

Next, we identified the interaction between circPLCE1-411 and HSP90α/RPS3 complex by immunoprecipitation in HCT8 and DLD1 cells (Fig. 5A). We found that RPS3 protein level was decreased considerably with the increased expression of circPLCE1-411 (Fig. 5B, top). However, the abundance of RPS3 mRNA levels did not change with the increase of circPLCE1-411 expression (Fig. 5B, bottom). This suggested that circPLCE1-411 may regulate RPS3 through a post-transcriptional mechanism. Treatment with the protein synthesis inhibitor cycloheximide (CHX) showed that the level of RPS3 protein degraded faster in HCT8 and DLD1 cells along with increased circPLCE1-411 expression, as compared to the control (Fig. 5C). In addition, treatment with the proteasome inhibitor MG132 attenuated the effect of circPLCE1-411 on the RPS3 protein level (Fig. 5D). Moreover, the RPS3 ubiquitylation level increased in cells with circPLCE1 overexpression. On the contrary, the ubiquitylation level of RPS3 decreased in cells with circPLCE1 knockdown (Fig. 5E).

It has been reported that the interaction between RPS3 and HSP90 enhances RPS3 stability by inducing resistance to proteasome-dependent degradation[32, 33]. However, dissociative RPS3 could bind to the chaperone-E3 ligase complex HSP70-CHIP to result in its ubiquitin-dependent degradation [34]. Therefore, we speculated that circPLCE1-411 bound to the HSP90α/RPS3 complex and promoted the separation of RPS3, resulting in the interaction between RPS3 and the HSP70-CHIP complex. To test this hypothesis, we transfected HEK293T cells with the same concentration of HA-HSP90α, His-RPS3 and Myc-HSP70 plasmids but increased the concentrations of Flag-circPLCE1 plasmids for subsequent immunoprecipitation assays. The results indicated that with the increased expression of circPLCE1-411, the interaction between RPS3 and HSP90α decreased, while the interaction between RPS3 and HSP70 increased (Fig. 5F). There is an ATP-binding domain in the N terminus of HSP90, which is essential for its chaperone activity [35, 36]. HSP90 inhibitors targeting its ATP-binding domain appear to dissociate RPS3 from HSP90, resulting in degradation of the free forms of RPS3 [32]. To identify the domain(s) of HSP90α responsible for the interaction between RPS3 and circPLCE1-411, we generated HSP90α truncation mutants and found that the interaction between RPS3 and circPLCE1-411 depended on the N-terminus of HSP90α (Fig. 5G).

As RPS3 is an important regulator of NF-κB, we further analyzed the impact of circPLCE1 on NF-κB signaling [31]. Western blot showed that circPLCE1-411 overexpression resulted in decreased p-P65 expression in the whole-cell extracts and downregulated P65 expression in the nucleus. As expected, the DNA-binding activity of nuclear P65 was also attenuated. On the contrary, circPLCE1-411 knockdown upregulated the protein level of p-P65 in the whole-cell extracts, increased the accumulation of P65 in the nucleus and strengthened the DNA-binding activity of nuclear P65 (Fig. 5H-I). Collectively, these data indicated that circPLCE1-411 bound to the N terminus of HSP90α to promote the dissociation of RPS3 from the HSP90α/RPS3 complex, resulting in the ubiquitin-dependent degradation of RPS3 to inhibit NF-κB signaling.

### circPLCE1-411 inhibits CRC growth and metastasis in vivo

**Fig. 6:**
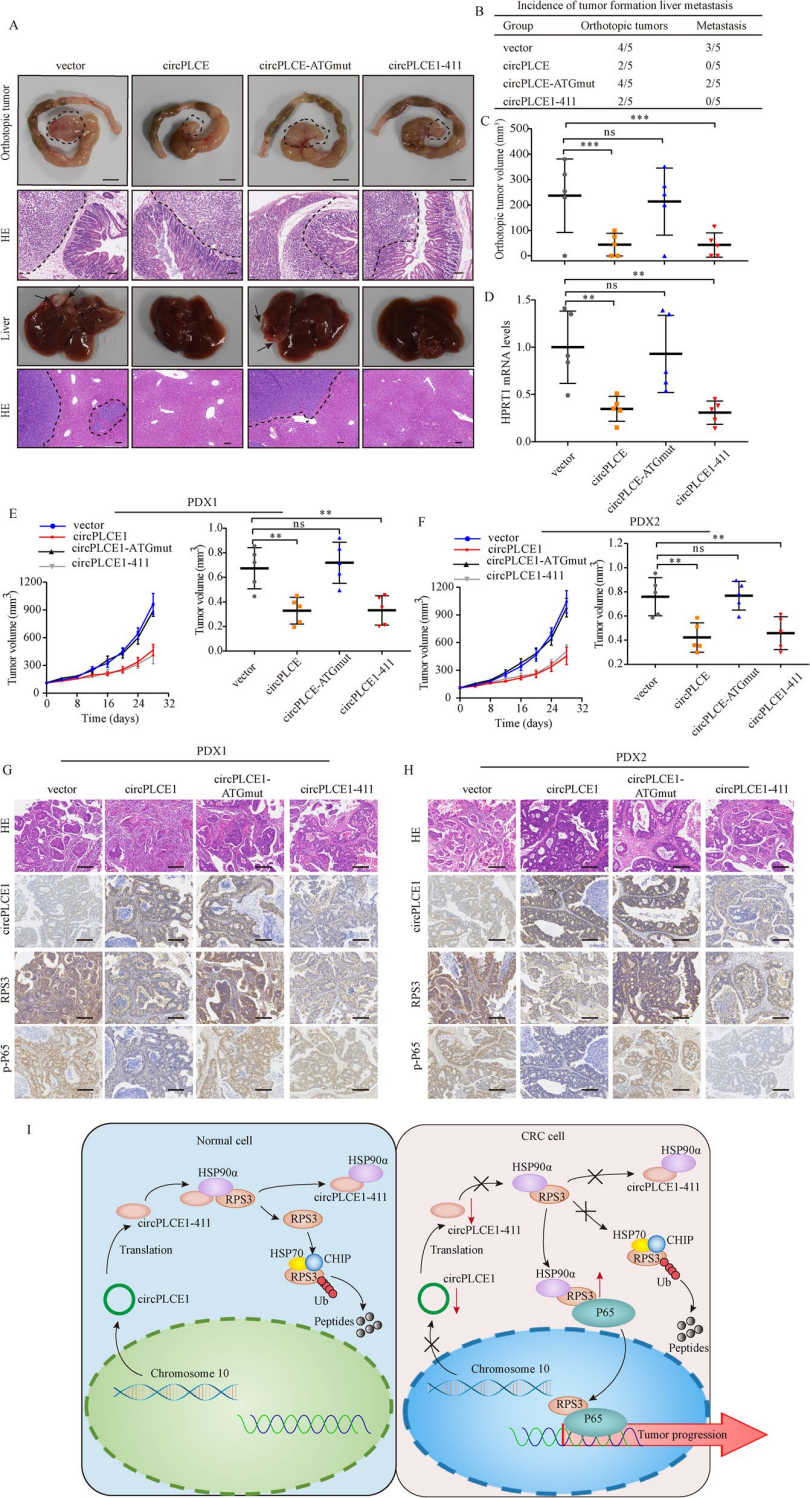
circPLCE1 inhibits CRC cell proliferation and metastasis in vivo*.***A** Representative images of gross inspection and H&E staining of the CRC orthotopic tumors or liver metastasis tumors (n = 5 per group). Orthotopic tumors scale bars = 0.5 cm, H&E scale bars = 100 μm. **B** Orthotopic xenograph CRC tumor formation and liver metastasis analysis. **C** Statistical analysis of orthotopic tumor volumes. **D** qRT-PCR analysis of human HPRT mRNA relative to mouse 18S rRNA in the livers of orthotopic xenograft CRC models. **E** and **F** Growth curves and tumor weight of PDX model tumors after treatment with vector, circPLCE1, circPLCE-ATGmut or circPLCE1-411 lentivirus (n = 5 per group). **G** and **H** H&E, circPLCE1, RPS3 and p-P65 staining in PDX model tumors mentioned above. scale bars = 200 μm. Values are represented as mean ± SD. *p < 0.05, **p < 0.01, ***p < 0.001, ns (no significance), by one-way ANOVA

To confirm the in vivo function of circPLCE1-411, we established orthotopic xenograft tumor models by injecting HCT8 cells transfected with the previously mentioned plasmids to observe the tumor growth and liver metastasis. Our results showed that the vector group and circPLCE-ATGmut group both exhibited 80% (4/5) colorectal tumor formation, with 60% (3/5) and 40% (2/5) liver metastasis, respectively. In the circPLCE1 and circPLCE1-411 groups, 2 of the 5 mice (40%) formed orthotopic tumors with smaller tumor sizes and no liver metastasis was identified (Fig. 6A-C). Furthermore, we analyzed the expression of human HPRT mRNA in the livers and confirmed that circPLCE1-411 induced a significant increase in the tumor burden of liver metastases (Fig. 6D). To further explore these effects, we applied two PDX models to evaluate the potential therapeutic benefits of circPLCE1-411 through intratumoral lentivirus injection. The results showed that treatment with circPLCE1 or circPLCE1-411 lentivirus led to significantly slower tumor growths than tumors treated with vector and circPLCE-ATGmut lentivirus (Fig. 6E and F). Moreover, we isolated the PDX tumors and assessed them by H&E, ISH and IHC staining, and found that circPLCE1 expression was upregulated in the circPLCE1 and circPLCE-ATGmut groups. In addition, the expression of RPS3 and p-P65 were reduced in circPLCE1 or circPLCE1-411 group (Fig. 6G and H). Taken together, these results identified the crucial roles of circPLCE1-411 in CRC tumor growth and metastasis in vivo.

## Discussion

In CRC, NF-κB signaling is constitutively active and plays important roles in CRC development and progression [4, 5]. Recent studies have shown that excessive activation of NF-κB was associated with advanced tumor stage and poor overall survival [7, 8]. It has been established that circRNAs are important regulators of cellular biological processes [20]. At present, most studies investigating circRNAs were limited to their roles as microRNA/protein sponges [21, 22]. However, recent studies showed that protein-coding circRNAs possessed important biological functions in the progression of CRC [24, 26]. Despite of this, the specifics of protein-coding circRNAs involved in NF-κB signaling regulation remain unclear. In this study, a novel protein circPLCE1-411 encoded by the circRNA circPLCE1 was identified as a crucial player in NF-κB activation in CRC. Mechanistically, circPLCE1-411 promoted the ubiquitin-dependent degradation of the critical NF-κB regulator RPS3 via directly binding to the HSP90α/RPS3 complex. This in turn promoted the dissociation of RPS3 from the complex, thereby reducing NF-κB nuclear translocation and inhibiting tumor progression.

circRNAs are aberrantly expressed in CRC and participate in a variety of biological processes, including tumor proliferation, metastasis and the remodeling of tumor microenvironment [37–39]. For example, circCAMSAP1 promotes CRC cells growth via the miR-328-5p/E2F1 axis [37]. circSPARC enhances the proliferation and migration of CRC by sponging miR-485-3p to upregulate JAK2 expression and recruiting FUS to facilitate the nuclear translocation of p-STAT3 [40]. circPPP1R12A encodes circPPP1R12A-73aa to promote colon cancer progression through activating Hippo-YAP signaling pathway [26]. circ0020397 facilitates the immune evasion of CRC by increasing the expression of PD-L1 and TERT genes, while circ100783 facilitates T cell ageing [41, 42]. In this study, we identified a novel circRNA, circPLCE1, which restrains CRC progression by encoding circPLCE1-411 which inhibits NF-κB activity.

Phospholipase C (PLC) family consists of 6 members, including PLCβ, γ, δ, ε, η and ξ with the enzyme activity to regulate membrane phospholipid metabolism to respond to hormones and growth factor stimulation [28]. Of the PLC family, PLCε has the largest size and has been extensively studied since its discovery in 1998 [43]. However, the role of PLCε in cancer progression is controversial because its function varies depending on the type of tumor [30]. In esophageal squamous cell carcinoma (ESCC), PLCE1 expression was upregulated and associated with poor overall survival and resistance to chemotherapy [44]. Moreover, the correlation between the single nucleotide polymorphism (SNP) of PLCE1 gene and ESCC was also identified[45, 46]. Besides ESCC, PLCE1 was identified as an oncogene in skin cancer, gallbladder cancer and prostate cancer [47–49]. However, PLCE1 is reported to be downregulated in CRC tissues and functions as a tumor suppressor [50]. Moreover, DNA methylation and histone deacetylation play vital roles in PLCE1 gene expression [51]. In this study, we found that circPLCE1 was downregulated in CRC with the same expression pattern as its cognate linear mRNA expression. We further deciphered the mechanisms for circPLCE1 to act as a tumor suppressor in CRC. Our results showed that circPLCE1 possessed the protein encoding ability to translate circPLCE1-411, which could regulate HSP90α/RPS3 complex to inhibit NF-κB signaling pathway.

Ribosomal protein S3 (RPS3) is a component of the 40S ribosomal subunit and has been reported to possess several extra ribosomal functions [52]. Recently, RPS3 was found to function as a specific component in NF-κB complexes. RPS3 could bind to the p65 subunit of NF-κB to promote nuclear accumulation of the NF-kB complex and enhance the expression of a number of target genes [53]. The biological function of RPS3 is regulated by post-transcriptional modifications, including phosphorylation and ubiquitination. For example, S6/T221 phosphorylation by protein kinase C-δ and T42 phosphorylation by Erk1 are required for the DNA repair function or nuclear translocation of RPS3 [54, 55]. The molecular chaperones, HSP90 and HSP70, play a key role in RPS3 ubiquitination. The interaction between RPS3 and HSP90 results in resistance to proteasome-dependent degradation of RPS3, thereby enhancing RPS3 stability [32]. However, dissociative RPS3 could bind to the chaperone-E3 ligase complex HSP70-CHIP to induce its ubiquitin-dependent degradation [34]. HSP90 could assist the recruitment or release of client proteins by its ATP-binding domain in the N-terminus[35, 56]. HSP90 inhibitors targeting the ATP-binding domain of HSP90 appeared to dissociate RPS3 from HSP90 and resulted in the degradation of the free forms of RPS3 [32]. In this study, we found a novel protein that regulated the HSP90α/RPS3 complex. We identified that circPLCE1-411 bound to the N-terminus of HSP90α to promote the dissociation of RPS3 from the HSP90α/RPS3 complex, leading to the ubiquitin-dependent degradation of RPS3.

NF-κB plays diverse roles at different stages of CRC development [6]. Therefore, it offers exciting opportunities which can be exploited clinically. There are several active clinical trials to assess the efficiency of NF-κB inhibitor the management of CRC [57–59]. Curcumin, a polyphenol derived from the spice turmeric, has been identified to inhibit tumor progression both in vivo and in vitro by targeting NF-κB signaling pathway [6, 60]. Moreover, Curcumin has also been shown to overcome chemo- and radio-resistance by blocking NF-κB activity [57]. With the development of RNA-related technologies, there is growing interest in developing novel RNA-based therapeutics [61]. In this study, we found that circPLCE1 encoded a novel protein to inhibit NF-κB activity to suppress CRC progression, which may enable circPLCE1 as a novel and potentially valuable RNA therapeutic target for CRC patients.

## Conclusions

Our findings revealed that circPLCE1-411 encoded by circPLCE1 could bind to the HSP90α/RPS3 complex to promote the dissociation of the critical NF-κB regulator RPS3 from the complex, thereby promoting its ubiquitin-dependent degradation, which ultimately resulted in the reduction of NF-κB nuclear translocation (Fig. 6I). Moreover, circPLCE1 presents an epigenetic mechanism that disrupts NF-κB nuclear translocation and can serve as a novel and promising therapeutic target and prognostic marker.

## Supplementary Information

**Additional file 1.**

## Data Availability

The RNA sequencing data used in the study (GSE163868) are available in a public repository from NCBI (https://www.ncbi.nlm.nih.gov/geo/query/acc.cgi?acc=GSE1 63868). The mass spectrometry data have been deposited to the ProteomeXchange Consortium via the PRIDE partner repository [62] with the dataset identifier PXD023617.

## Abbreviations

CRC: Colorectal carcinoma
NF-κB: Nuclear factor-κB
circRNAs: Circular RNAs
ORFs: Open reading frames
HPRT: Human hypoxanthine phosphoribosyltransferase
qRT-PCR: Quantitative real-time PCR
PLCE: Phospholipase C epsilon-1 gene
FISH: Fluorescence in situ hybridization
PDOs: Patient-derived organoids
shRNAs: Short hairpin RNAs
IRES: Internal ribosome entry site
CHX: Cycloheximide
SNP: Single nucleotide polymorphism
RPS3: Ribosomal protein S3
