# Supplementary material for: A novel NF-κB regulator encoded by circPLCE1 inhibits colorectal carcinoma progression by promoting RPS3 ubiquitin-dependent degradation
Source: Mol Cancer. 2021 Aug 19;20:103. doi: 10.1186/s12943-021-01404-9 (PMC8375079; doi:10.1186/s12943-021-01404-9)
Supplement: Supplementary file 1 — Additional file 1. [file 12943_2021_1404_MOESM1_ESM.pdf]

Title: A novel NF-κB regulator encoded by circPLCE1 inhibits colorectal carcinoma progression by promoting RPS3 ubiquitin-dependent degradation

Authors: Zhen-xing Liang[1, 2, 3], Hua-shan Liu[1, 2, 3], Li Xiong[4], Xin Yang[1], Feng-wei Wang[5], Zi-wei Zeng[1], Xiao-wen He[1], Xian-rui Wu[1, 2, 3] and Ping Lan[1, 2, 3]

From: [1]Department of Colorectal Surgery, The Sixth Affiliated Hospital, Sun Yat-sen University, Guangzhou, Guangdong, China.

[2]Guangdong Provincial Key Laboratory of Colorectal and Pelvic Floor Diseases, The Sixth Affiliated Hospital, Sun Yat-sen University, Guangzhou, Guangdong, China.

[3]Bioland Laboratory, Guangzhou Regenerative Medicine and Health Guangdong Laboratory, Guangzhou, China.

[4]Department of Endocrinology, The First Affiliated Hospital of Sun Yat-sen University, Guangzhou, China.

[5]State Key Laboratory of Oncology in South China, Sun Yat-sen University Cancer Center, Guangzhou, Guangdong, China.

Authorship: Zhen-xing Liang, Hua-shan Liu and Li Xiong contributed equally to this study.

Short Title: A novel NF-κB regulator promotes RPS3 ubiquitin-dependent degradation

Correspondence authors:

Ping Lan and Xian-rui Wu

The Sixth Affiliated Hospital, Sun Yat-sen University

26 Yuancun Erheng Rd, Guangzhou, Guangdong, China, 510655

Tel: 086-020-38255801; Fax: 011-86-20-38254166.

Email: lanping@mail.sysu.edu.cn; wuxianr5@mail.sysu.edu.cn

Conflict of interest: The authors have declared that no conflict of interest exists.

Supplemental Figures

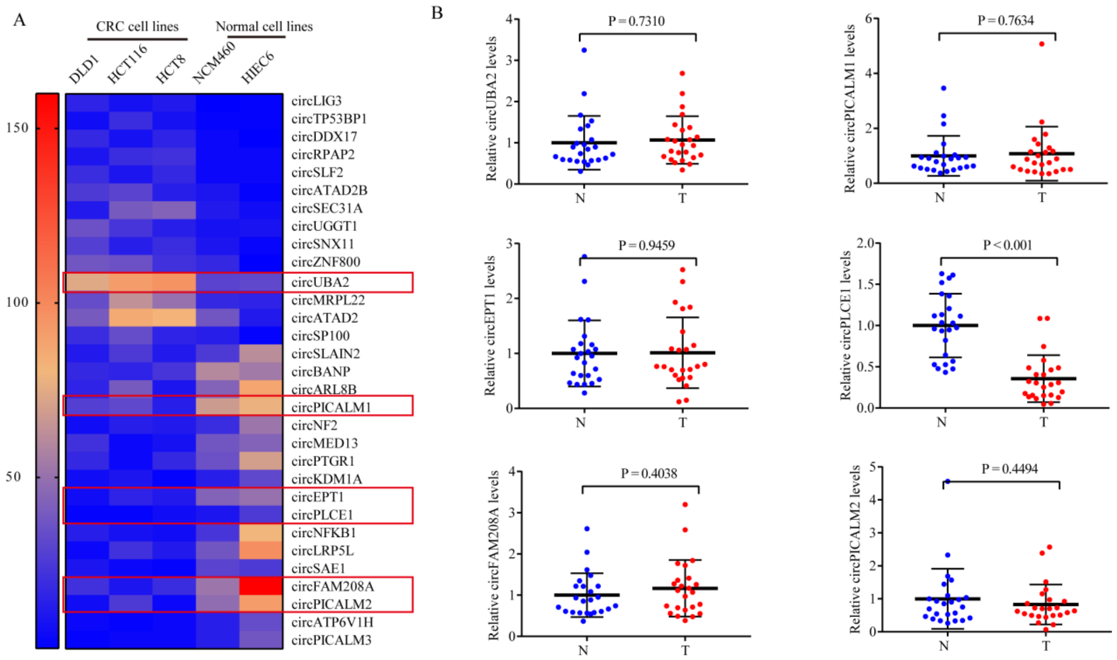

Figure S1. circRNAs expression

A Heatmap of the screening circRNAs with with fold change > 2 or <0.5, p <0.05 and transcript abundance >0 sequence between normal human intestinal epithelial cell lines and CRC cell lines. **B** qRT-PCR analysis of six candidate circRNAs in 24 paired CRC samples and normal adjacent tissues. N, normal adjacent tissues tissues; T, tumor tissues. Values are represented as mean ± SD, by 2-tailed Student’s t test.

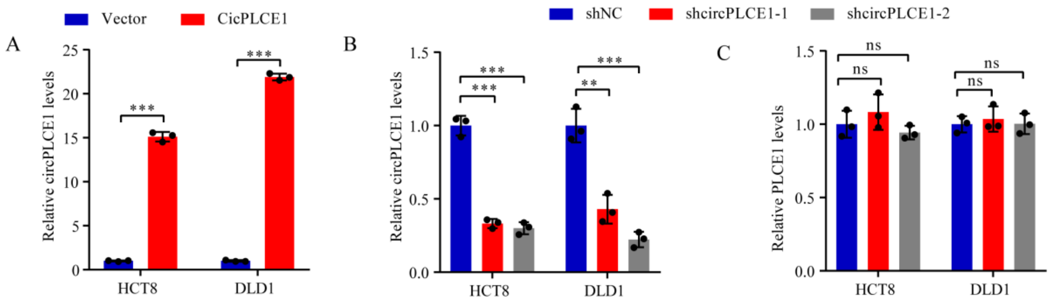

Figure S2. circPLCE1 expression in circPLCE1 overexpressed or knockdown CRC cells

A qRT-PCR analysis of circPLCE1 expression in circPLCE1 overexpressed HCT8 and DLD1 cells. **B** qRT-PCR analysis of circPLCE1 expression in circPLCE1 knockdown HCT8 and DLD1 cells. **C** qRT-PCR analysis of linear PLCE1 mRNA expression in circPLCE1 overexpressed or knockdown HCT8 and DLD1 cells. Values are represented as mean ± SD. $^{**}p < 0.01$, $^{***}p < 0.001$, ns (no significance), by 2-tailed Student's t test (**A** and **B**) and one-way ANOVA (**C**).

A

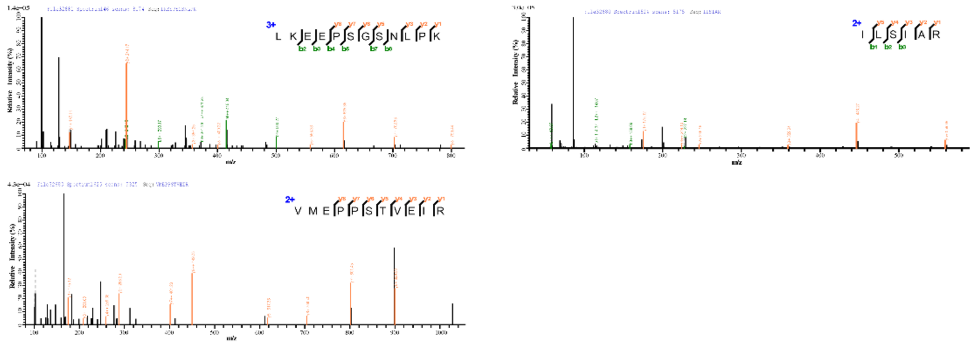

B circPLCE1-411 411aa

MTSEEMTASVLIPVTQRKVVSAQSAADESSEKVSDINISKAHTVRRSGETSHTISQLNKLKEEPSGSNLPKILSIAREKIVSDENSNEKCWEKIMPDSAKNLNINCNNILRNHQHGLPQR
QFYEMYNSVAEEDLCLETGIPSPLERKVFPGIQLELDRPSMGISPLGNQSVIIETGRAHPDSRRAVFHFHYEVDRRMSDTFCTLSENLILDDCGNCVPLPGGEEKQKKNYVAYTCKLME
LAKNCDNKNEQLQCDHCDTLNDKYFCFEGSCEKVDMVYSGDSFCRKDFTDSQAAKTFLSHFEDFPDNCDDVEEDAFKSKKERSTLLVRRFCKNDREVKKSVYTGTRAIVRTLPSG
HIGLTAWSYIDQKRNGPLLPCGRVMEPPSTVEIRQDGSQRLSEAQWYPETGIIQTGF

PLCE1 (NP_057425.3) 2302 aa

MTSEEMTASVLIPVTQRKVVSAQSAADESSEKVSDINISKAHTVRRSGETSHTISQLNKLKEEPSGSNLPKILSIAREKIVSDENSNEKCWEKIMPDSAKNLNINCNNILRNHQHGLPQR
QFYEMYNSVAEEDLCLETGIPSPLERKVFPGIQLELDRPSMGISPLGNQSVIIETGRAHPDSRRAVFHFHYEVDRRMSDTFCTLSENLILDDCGNCVPLPGGEEKQKKNYVAYTCKLME
LAKNCDNKNEQLQCDHCDTLNDKYFCFEGSCEKVDMVYSGDSFCRKDFTDSQAAKTFLSHFEDFPDNCDDVEEDAFKSKKERSTLLVRRFCKNDREVKKSVYTGTRAIVRTLPSG
HIGLTAWSYIDQKRNGPLLPCGRVMEPPSTVEIRQDGSQRLSEAQWYPIYNAVRREETENTVGSLLHFLTKLPASETAHGRISVGPCLKQCVRDTVCEYRATLQRTSISQYITGSLLEAT
TSLGARSGLLSTFGGSTGRMMLKERQPGPSVANSNALPSSSAGISKELIDLQPLIQFPEEVASILMEQEQTIYRRVLPVDYLCFLTRDLGTPECQSSLPCLKASISASILTTQNGEHNALED
LVMRFNEVSSWVTWLILTAGSMEEKREVFSYLVHVAKCCWNMGNYNAVMEFLAGLRSRKVLKMWQFMDQSDIETMRSLKDAMAQHESSCEYRKVVTRALHIPGCKVVPFCGVF
LKELCEVLDGASGLMKLCPRYNSQEETLEFVADYSGQDNFLQRVGQNGLKNSEKESTVNSIFQVIRSCNRSLETDEEDSPSEGNSSRKSSLKDKSRWQFIIGDLLDSDNDIFEQSKEYD
SHGSEDSQKAFDHGTELIPWYVLSIQADVHQFLLQGATVIHYDQDTHLSARCFLQLQPDNSTLTWVKPTTASPASSKAKLGVLNNTAEPGKFPLLGNAGLSSLTEGVLDLFAVKAVY
MGHPGIDIHTVCVQNKLGSMFLSETGVTLLYGLQTTDNRLLHFVAPKHTAKMLFSGLLELTRAVRKMRKFPDQRQQWLRKQYVSLYQEDGRYEGPTLAHAVELFGGRRWSARNPSP
GTSAKNAEKPNMQRNNTLGISTTKKKKKILMRGESGEVTDDEMATRKAKMHKECRSRSGSDPQDINEQEESEVNAIANPPNPLPSRRAHSLTTAGSPNLAAGTSSPIRPVSSPVLSSSN
KSPSSAWSSSSWHGRIKGGMKGFQSFMVSDSNMSFVEFVELFKSFSVRSRKDLKDLFDVYAVPCNRSGSESAPLYTNLTIDENTSDLQPDLDLLTRNVSDLGLFIKSKQQLSDNQRQIS
DAIAAASIVTNGTGIESTSLGIFGVGILQLNDFLVNCQGEHCTYDEILSIIQKFEPSISMCHQGLMSFEGFARFLMDKENFASKNDESQENIKELQLPLSYYYIESSHNTYLTGHQLKGESS
VELYSQVLLQGCRSVELDCWDGDDGMPIIYHGHTLTTKIPFKEVVEAIDRSAFINSDLPIIISIENHCSLPQQRKMAEIFKTVFGEKLVTKFLFETDFSDDPMLPSPDQLRKKVLLKNKKL
KAHQTPVDILKQKAHQLASMQVQAYNGGNANPRPANNEEEEDEEDEYDYDYESLSDDNILEDRPENKSCNDKLQFEYNEEIPKRIKKADNSACNKGKVYDMELGEEFYLDQNKKE
SRQIAPELSDLVIYCQAVKFPGLSTLNASGSSRGKERKSRKSIFGNNPGRMSPGETASFNKTSGKSSCEGIRQTWEESSSPLNPTTSLSAIIRTPKCYHISSLNENAAKRLCRRYSQKLTQH
TACQLLRTYPAATRIDSSNPNPLMFWLHGIQLVALNYQTDDLPLHLNAAMFEANGGCGYVLKPPVLWDKNCPMYQKFSPLERDLDSMDPAVYSLTIVSGQNVCPSNSMGSPCIEVDV
LGMPLDSCHFRTKPIHRNTLNPMWNEQFLFHVHFEDLVFLRFAVVENNSSAVTAQRIIPLKALKRGYRHLQLRNLHNEVLEISSLFINSRRMEENSSGNTMSASSMFNTEERKCLQTHR
VTVHGVPGPEPFTVFTINGGTKAKQLLQQILTNEQDIKPVTTDYFLMEEKYFISKEKNECRKQPFQRAIGPEEEIMQILSSWFPEEGYMGRIVLKTQQENLEEKNIVQDDKEVILSSEEE
SFFVQVHDVSPEQPRTVIKAPRVSTAQDVIQQTLCKAKYSYSILSNPNPSDYVLLEEVVKDTTNKKTTTPKSSQRVLLDQECVFQAQSKWKGAGKFILKLKEQVQASREDKKKGISFA
SELKKLTKSTKQPRGLTSPSQLLTSESIQTKEEKPVGGLSSSDTMDYRQ

C

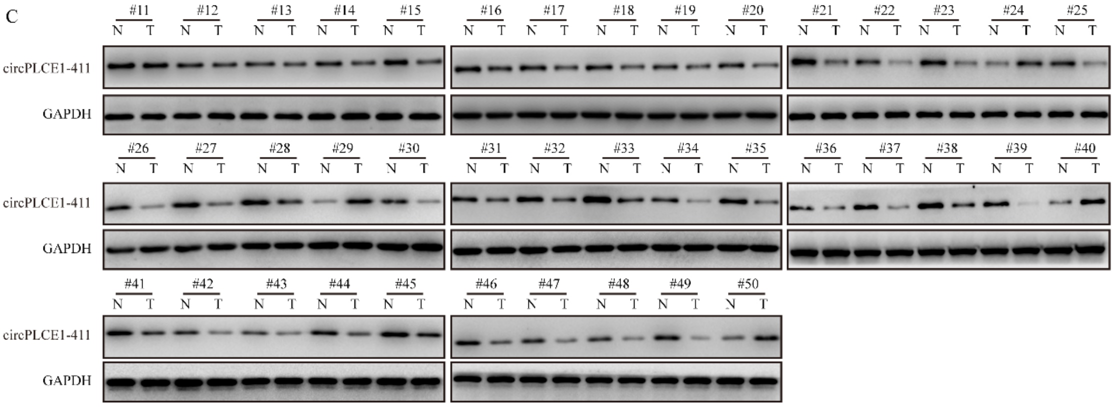

Figure S3. circPLCE1-411 identification and expression in CRC tissues

A circPLCE1-411 amino acids identified by MS. **B** circPLCE1-411 sequence was compared with the PLCE1 full length sequence, and the underlined peptide sequence was shared by circPLCE1-411 and PLCE1. Peptide sequences identified by MS were highlighted in yellow and the unique amino acid sequence of circPLCE1-411 was marked in red. **C** Western blot analysis of circPLCE1-411 expression in paired CRC samples and normal adjacent tissues with PLCE1 antibodies.

Supplemental Tables

Table S1 Correlation between circPLCE1 expression and clinicopathologic characteristics of CRC patients

Characteristics	Frequency	circPLCE1 expression level		
		Low	High	*p*-value[a]
Gender				0.892
Female	118	49	69	
Male	144	61	83	
Age				0.176
≤59	120	45	75	
>59	142	65	77	
T stage				< 0.001
T1+T2	78	11	67	
T3+T4	184	99	85	
N stage				< 0.001
N0	176	49	127	
N1+N2	86	61	25	
M stage				< 0.01
M0	253	98	155	
M1	9	8	1	
Clinical stage				< 0.001
I+II	176	49	127	
III+IV	86	61	25	

[a] Chi-square test

Table S2 Conservative analysis

```
                                                                         Stop
Mus_musculus_(house_mouse)              -GgcACAGaAAACAcTCAAACTGGATTTTTAAATCccTGCAcATtAacaATa-agcCAcA
Rattus_norvegicus_(Norway_rat)          -GgAACAGaAAACAcTCAAACTGGATTTTTAAATCATTGCAcATtAacGATacaAcCccA
Sus_scrofa_(pig)                        ------AGGAAACATTCAgACTaGATTTTTAAATCATTGtAAgcCAGTGATG-GATCAGA
Homo_sapiens_(human)                    -GAAACAGGAAtCATTCAAACTGGATTTTaAAATCATTGCAAATCAGTGATG-GATCAGA
Macaca_mulatta_(Rhesus_monkey)          gGAAACAGGAAtCATTCAAACTGaATTTTaAAATCATTGCAAATCAGTGATG-GATCAGA
                                                                          TAA

Mus_musculus_(house_mouse)              AGTcGCAGAATGtgATCCCGAAccAAAtcCTTCtAAAAA-TTAGCTTCgGGcAACAGAGa
Rattus_norvegicus_(Norway_rat)          AGTTGCAGAATGtgAcCttGAAcAAAAtcCTTCtAAAAAATTAGCTTCAGGcAACAGAGa
Sus_scrofa_(pig)                        AGTTaCAGAATGCAcTgCCtAATAAAAGTCTTCAAAAgAATT-GCTTCAaaTAACcaAaG
Homo_sapiens_(human)                    AGTTGCAGAgTGCAATCCCGAgTAAAAGTCTTCAAAAAAtTTtGCTTCAGGTAACAGAGG
Macaca_mulatta_(Rhesus_monkey)          AGTTGCAGAgTGCAATCCCGAgTAAAAGTCTTCAAAAAttTTtGCTTCAGGTAACAGAGG

Mus_musculus_(house_mouse)              AaagAAGAAAAcgGAcCTgCtgttTTgAAAtaCTGgTgTAaAAcAAACT----GTtcTtG
Rattus_norvegicus_(Norway_rat)          A---AAGAAAAgTGAaCTgttgttTTgAAAtaCTGgTgTAaAAcAAACT----GTCcTtG
Sus_scrofa_(pig)                        A---AAGAAAATTGATCTACCACCTTAAAACCtTGATCTAGAAAAAcaT--TgGaCATGG
Homo_sapiens_(human)                    A---AgGAAAATTGATCTACCACCTTAAAACCCTGATCTAGAAAAAAtaTATAtTCATGa
Macaca_mulatta_(Rhesus_monkey)          A---AgGAAAATTGATCTACCACCTTAAAACCCTGATCTAGAAAAAAtaTATAtTCATGa

Mus_musculus_(house_mouse)              TAGGAAaTTACAACTGGaAAgtTTTcTTTGGCgCTTAATaTACCTGAATGAAAGGAAcTG
Rattus_norvegicus_(Norway_rat)          TAGGAAaTTACAACTGGaAAAtTTTgTTTGGCTCTTAATaTAtCTGAATGAAAGGAAcTG
Sus_scrofa_(pig)                        TAGGAAGTaAtgACTtGGgAAATTTATTTGGCTCTTAATGTgCCTaAATGAgAGGAATTG
Homo_sapiens_(human)                    TAGGAAGTTAtAACTaaGAAAATTTATTTGcCTCTTAATGctCCTGAATGAAAGGAATTa
Macaca_mulatta_(Rhesus_monkey)          TAGaAAGTTACAACTGaGAAAATTTATTTGcCTCTcAATGctCCTGAATGAAAGGAATTa

Mus_musculus_(house_mouse)              TCAagcTTGcTTCgCAcaAaGACTgGaATTATCTGAGATTAT------------------
Rattus_norvegicus_(Norway_rat)          TCAC-cTTacTTCTCAc---GACTgGaATTATCTGAGATTAT------------------
Sus_scrofa_(pig)                        TCAC-TTTGTTTCTCAG-AGtACTTGTA-TATCTGAGATTATTtTAATAATCAGTgATTT
Homo_sapiens_(human)                    TCcC-TTTGTTctTtgGGAGGACTTGTg-TATCTGAGATTgTTGTAATAATCAGTcATTT
Macaca_mulatta_(Rhesus_monkey)          TCcC-TTTGTTcCTtgGGAGGACTTGTg-TATCTGAGATTgTTGTAATAATCAaTaATTT
                                                      Start
Mus_musculus_(house_mouse)              --cTgttgTCTgGACATGATCgCCAGGGAaGgGAACACAGAagAgTAGctgAAtgAacgA
Rattus_norvegicus_(Norway_rat)          --cTgttgTCTgGACATGATCgCCAGGGAaGgGAACACAGAagAgTAG-CAActCAaGgA
Sus_scrofa_(pig)                        TATTAAcATCTTGACATGATCACaAGGGAGGAGAA-ACAaAGCAATAGTtAAAACtTGct
Homo_sapiens_(human)                    TATTAAAAcCTTGACATGATCACCAGGGAGGAaAA-AtAGAGCAATAGTCAAAACcTGtg
Macaca_mulatta_(Rhesus_monkey)          TATTAAAAcCTTGACATGATCAtCAGGGAGGAGAA-AtAGAGCAATAGTCAAAACcTGtg
                                                      ATG
Mus_musculus_(house_mouse)              CagTGGT-CAAGATGACgTCgGAAGAAATGGCcGCTTCTGTcCTCATcCCTGTGACTCAa
Rattus_norvegicus_(Norway_rat)          CGcTGGT-CAAGATGACgTCcGAAGAAATGGCcGCTTCctTcCTCATcCCgGTGcCTCAa
Sus_scrofa_(pig)                        CaTTGaT-CAAGATGACTTCTGAAGAAATGGCAGCTTCTGTTCTCtTACCTGTGACTCAG
Homo_sapiens_(human)                    tGTTaGTCCAAGATGACTTCTGAAGAAATGaCAGCTTCTGTTCTCATACCTGTGACTCAG
Macaca_mulatta_(Rhesus_monkey)          tGTTaGTCCAAGATGACTTCTGAAGAAATtaCAGCTTCTGTTCTCATACCTGTGACTCAG

Mus_musculus_(house_mouse)              AGgAAAGTGGcTTCTGCCCAGTCGGtgGCAGAaGAgAGgAGTGtAAAGGTCTCAGAtgcg
Rattus_norvegicus_(Norway_rat)          AGgAAAGTGGcTTCTGCCCAGTCaGtgGCAGAgGAgcGggGTGAAAAGGTCTCAGAagcC
Sus_scrofa_(pig)                        AGAAAAGTGGTaTCTGCtCAGTtGGCTGtAGATGAAAGTAGTGAAAAGGgCaCAGACATC
Homo_sapiens_(human)                    AGAAAAGTGGTTTCTGCCCAGTCGGCTGCAGATGAAAGTAGTGAAAAGGTCTCAGACATC
Macaca_mulatta_(Rhesus_monkey)          AGAAAAGTGGTTTCTGCCCAGTCaGCTGCAGATGAAAGTAGTGAAAAGGTCTCAGACATC

Mus_musculus_(house_mouse)              gggATTCCtcgGGCACgTgCTGgCAGgCAGgGTGcGctcAtccCTCcTACCATCTCACAg
Rattus_norvegicus_(Norway_rat)          ggcATTCCtAAGaCACgagCTGgCAGACAGgGTGGGctcACccCTCgTACCATCTCACAg
Sus_scrofa_(pig)                        AATtTTCCAAAGtCcCtTcCcaTCAGgCAGtGTGGaGAGACTTCTCATACCATCTCACAA
Homo_sapiens_(human)                    AATATTtCAAAaGCACATACTGTCAGACgaAGTGGGGAGACTTCTCATACCATCTCACAA
Macaca_mulatta_(Rhesus_monkey)          AATATTtCAAAaGCACATACTGTCAGACAaAGTGGGGAGACTTCTCATACCATCTCACgA

Mus_musculus_(house_mouse)              tgGAACAAACaTAAgGAAGAgTCgTCTaGAAGtgAtTTGtCCAAGgTctTCTCAATAGCG
Rattus_norvegicus_(Norway_rat)          CgGAACgAACcTgAgGAAGAgTCTcCTaGAActgAtTTttCCcAGgTctTCTCAATAGCG
Sus_scrofa_(pig)                        CTGAACAcACTTAcAGAAGAATCTTCTGGAAGCAACTTGCaCAcGAaTCTCTCAgcAGCa
Homo_sapiens_(human)                    CTGAACAAACTTAAAGAAGAAcCTTCTGGAAGCAACTTGCCaAAGATTCTCTCAATAGCG
Macaca_mulatta_(Rhesus_monkey)          CTGAACAAACTTAAAGAAGAAcCTTCTGGAAGCAACTTGCCaAAGATTCTCTCAATAaCa
```

```
Mus_musculus_(house_mouse)         AGGGgGgAAcTAGTctGcGATGAGAAttccAAcGAAgAGgGCTGGGAGgAAAACgcaCCg
Rattus_norvegicus_(Norway_rat)     AGGGgGgAAcTAGacAGcGAcGAGAAtcacAATGAAAgGTGCTGGGAGgAAAAtgTGCCA
Sus_scrofa_(pig)                   AaGGAGAcAATAaTGAGTGATGAGAACAGTAATGAAAAGTGCTGGGAGAgAAgCATGCCA
Homo_sapiens_(human)               AGGGAGAAAATAGTGAGTGATGAGAACAGTAATGAAAAaTGtTGGGAGAAAAtCATGCCA
Macaca_mulatta_(Rhesus_monkey)     AGGGAGAAAATAGTGAGTGATGAGAACAGTAATGAAAAaTGCTGGGAGAAAAgCATGCCA

Mus_musculus_(house_mouse)         GAcTCccCGgAAAACCacgcgATgAACgGCAACAgttTAgTGcAAAgCCAcCAGCAccag
Rattus_norvegicus_(Norway_rat)     GgTTCcaCGAAAAACCacgcggTTAACTGCAACAgCtTATTGcAAAgCCAcCAGCATGcg
Sus_scrofa_(pig)                   GATTCTGtGAAAAACCTTAAtATTAACTGCAACAACATATTGAAAAAtCATCAGCgTGGC
Homo_sapiens_(human)               GATTCTGCGAAAAACCTTAAcATTAACTGCAACAACATATTGAgAAACCATCAGCATGGC
Macaca_mulatta_(Rhesus_monkey)     GATTCTGtGAAAAACCTTAAcATTAACTGCAACAACATATTGAgAAACCATCAGCATGGC

Mus_musculus_(house_mouse)         tTTCCcCgGAGCCAGcTTTgTGAAgcCcGtGACTCTGTCAC---aGAAGACCcGTGTTTg
Rattus_norvegicus_(Norway_rat)     CTTCCcCcGAGCCAGcTcTgTGAAgTCTGtGACTCTGTCACAGAaGAAcACCTGTGTTTg
Sus_scrofa_(pig)                   CTTCCTCAGAGCCAGTTTTATGAAAcaTGCGAgTCTaTCACAGAGGAAGACCTGTGTTTA
Homo_sapiens_(human)               CTTCCTCAGAGaCAaTTTTATGAAATgTaCaACTCTGTtgCtGAGGAAGACtTGTGTTTA
Macaca_mulatta_(Rhesus_monkey)     CTTCCTCAaAGaCAGTTTTATGAAATgTaCaACTCTGTtgttGAGGAAGACCTGTGTTTA

Mus_musculus_(house_mouse)         cAgcCTGGAATTCCTTCTCCcCTGGAAAGgAAGGTGcTCCCTGGAATTCAACTGGAgATG
Rattus_norvegicus_(Norway_rat)     cAgcCTGGgATTCCTTCTCCACTGGAAAGgAAGGTGTTtCCTGGgATcgAACTGGAgATG
Sus_scrofa_(pig)                   GAAACTGGAATcCCTTCTCCACTGGAAAGAAAGGTGTTCCCTGGAATTCAACTGGAAATG
Homo_sapiens_(human)               GAAACTGGAATTCCTTCTCCACTGGAAAGAAAGGTGTTCCCTGGAATTCAACTGGAAcTa
Macaca_mulatta_(Rhesus_monkey)     GAAACTGGAATTCtgTCTCCACTGGAAAGAAAGGTGTTCCCTGGAATTCAACTGGAAcTa

Mus_musculus_(house_mouse)         GAggactCTCCCATGGaCgTGAGcCCTgcgGGAAgTCAGcCtagGATCATgGAGtCcaGC
Rattus_norvegicus_(Norway_rat)     GAggactCTCCCATGGaCgTGAGcCCTTTgGGAAAcCAGcCtGgGATCATgGAGtCcaGC
Sus_scrofa_(pig)                   GACAGACCTCCCATGGGCATGgTCCaTTAGGAAcTCAaTCAGccATCATAGAGAtgGGC
Homo_sapiens_(human)               GACAGACCTtCCATGGGCATtAGTCCTTTAGGAAATCAGTCAGtGATCATAGAGACAGGC
Macaca_mulatta_(Rhesus_monkey)     GACAGACCTtCCATGGGCATtAaTCCTTTAGGAAATCAGTCAGcaATCATAGAGACAGGC

Mus_musculus_(house_mouse)         gGAcCtCACtCTGACcGCAACACGGCgGTATTTCAcTTcCATTATGAAGcTGACAGgAcA
Rattus_norvegicus_(Norway_rat)     gGAcCtCACtCTGACcGCAACAtGGCgGTATTTCATTTcCATTATGcAGgTGACAGgAcA
Sus_scrofa_(pig)                   cGgGCACACCCTGACAGCAACcCGaCAGTgaTTCAcTTTCgTTATGAAGTgGACAGAAGA
Homo_sapiens_(human)               AGAGCACACCCTGACAGCAgaAgGGCAGTATTTCATTTTCATTATGAAGTTGACAGAAGA
Macaca_mulatta_(Rhesus_monkey)     AGAGCAgACCCTGACAGCAgCACGGCAGTATTTCATTTTCATTATGAAGTTGACAGAAGA

Mus_musculus_(house_mouse)         ATGTCgGAtgCTTTtcaTACCCTGTCAGAAAACcTgATTTTGGATGAcTGTGcAAATTGT
Rattus_norvegicus_(Norway_rat)     ATGcCAGgtgCTTTCcaTACCCTGTCAGAAAAaTTcATTTTGGATGAcTGTGcAAAcTGT
Sus_scrofa_(pig)                   ATGcCAGACACTTTCTGTcCCCTGTCAGAtAACTTggTcTTGGATGATTGTGGAAAcTGT
Homo_sapiens_(human)               ATGTCAGACACTTTCTGTACCCTaTCAGAAAACTTAATTTTaGAcGATTGTGGAAATTGT
Macaca_mulatta_(Rhesus_monkey)     ATGTCAGACACTTTCTGTACCCTaTCAGAAAACTTAATTTTaGAcGATTGTGGAAATTGT

Mus_musculus_(house_mouse)         GTtACtCTtCCTGG------GGGGcAGCAAAAtAAAAATTgCATGGCGTATgCTTGCAAA
Rattus_norvegicus_(Norway_rat)     GTtACtCTtCCTGG------GGGGcAGCAAAAcAAAAATTACATGGCGTATACTTGCAAA
Sus_scrofa_(pig)                   GT---ACTgCCTGGtGtTGgtGGGgAGCcAAAGAAAAATTACtTGGCGTATACTTGCAAA
Homo_sapiens_(human)               GTAcCACTACCTGGGGGTGAGGaGAAGCAAAAGAAAAAcTAtgTGGCaTATACcTGtAAA
Macaca_mulatta_(Rhesus_monkey)     GTAcCACTACCTGGGGGTGAGGaGAAGCAAAAGgAAAAcTACATGGCaTATACcTGtAAA

Mus_musculus_(house_mouse)         CTGgTGGAgTTGaCAAgAAcCTGTGgaAgTAAGAATGGCAagTcCAGTGTGAgCAcTGT
Rattus_norvegicus_(Norway_rat)     CTGgTGGAgTTGaCAAgAAcCTGTGgTAgTAAGAATGGCAGCTGaAGTGTGAcCAcTGc
Sus_scrofa_(pig)                   CTGATGGAATTGGCAAAtAACTGTGATAATAAGAATaGGCAGCTGCAGTaTGATCcTTGT
Homo_sapiens_(human)               CTGATGGAATTGGCcAAAAAtTGTGATAATAAGAATGaGCAGCTGCAGTGTGATCATTGT
Macaca_mulatta_(Rhesus_monkey)     CTGATGGAATTGGCcAAAAAtTGTGATAATAAGAATGaaCAGCTGCAGTGTGATCATTGT

Mus_musculus_(house_mouse)         acCtCgcTGcgcGATgAATACcTGTGCTTcGAAaGCTCcTGCtcaAAGGCcGAtGaGGTc
Rattus_norvegicus_(Norway_rat)     acCtCgcTGcggGATgAATACcTGTGCTTTGAAaGCTCcTGCCgGAAGGCtGAgGctcTc
Sus_scrofa_(pig)                   GACcCtTTGAATGAcAAATcCTTGTGCTTTGAAGcCTCTTtCCcaAAGGCcaAtGTGcTA
Homo_sapiens_(human)               GACACcTTGAATGATAAATACTTtTGCTTTGAAGGCTCTTGtgAGAAGGttGAcaTGGTA
Macaca_mulatta_(Rhesus_monkey)     GACACcTTGAATGATAAATACTTtTGCTTTGAAGGCTCTTGCCAGAAGGtgGAcaTGGTA

Mus_musculus_(house_mouse)         TGcTCAGGTGgTgGCTTTTGCgAGGAcGgCTTTgCTcACgGTCCtgCTGCCAAGACtTTT
Rattus_norvegicus_(Norway_rat)     TccTCAGGTGgTgGCTTTTGCgAGGAcGgaTTTACTcAtgGTCCctCTGCCAAGACtTTT
Sus_scrofa_(pig)                   TGTTCAaGgGAcAGCTTTTGCAgGGAgGAtTTTACTGACAaTCCAcCTGCCAAGACCTTT
Homo_sapiens_(human)               TaTTCAGGTGATAGCTTTTGtAgGaAAGACTTTACTGACAGTCaAgCTGCCAAGACCTTT
Macaca_mulatta_(Rhesus_monkey)     TGTTCAaGTcATAGCTTTTGtAAGGAAGAtTTTACTGACAGTCaAaCTGCCAAGACCTTT

Mus_musculus_(house_mouse)         CTGAaCCcTcTgGAGGAtTTCtCTGAcAATTGTGAAGAcGT---AGAcGATtTTTTAAA
Rattus_norvegicus_(Norway_rat)     CTGAaCCcTTTgGAGGAaTTCtCTGATAATTGTGAAGAcGT---gGAcGATaTTTTAAA
Sus_scrofa_(pig)                   CTGAGCCATTTTGAGGACTTCCCTGATAATTGTGAAGATGTAGAAGAAGATtTTcTcAAA
Homo_sapiens_(human)               tTGAGCCATTTTGAGGACTTCCCTGATAATTGTGAtGATGTAGAAGAAGAcGcTTTTAAA
Macaca_mulatta_(Rhesus_monkey)     tTGAGtCATTTTGAGGAtTTCCCTGATAATTGTGAAGATGTAGAAGAAGATGcTTTTAAA
```

Antibody recognizes base sequence

```
Mus_musculus_(house_mouse)           AGCAAAAAGGAGCGGTCCACgTTGTTAGTCcGaAGgTTTTGTAAAAATGACAGgGAAGTT
Rattus_norvegicus_(Norway_rat)       gGCAAAAAGGAGCGGTCCACTTTGTTAGTCcGaAGgTTTTGTAAAAATGACAGgGAAGTT
Sus_scrofa_(pig)                     AGCAAAAgGGAGCGGTCCACTTgGTTAGTgAGGAGATTTTGTAAAAAcGACAGAGAAGTT
Homo_sapiens_(human)                 AGCAAAAAGGAGCGaTCCACTTTGTTAGTCAGGAGATTcTGTAAAAATGACAGAGAAGTT
Macaca_mulatta_(Rhesus_monkey)       AGCAAAAAGGAGCGGTCCACTTTGTTAGTCAGGAGATTcTGTAAAAATGACAGAGAAGTT

Mus_musculus_(house_mouse)           AAGAAgTCTGTGTATACTGGGACcAGAGCCATcaTGAGAACTCTGCCTTCTGGCtgtATT
Rattus_norvegicus_(Norway_rat)       AAGAAgTCTGTGTATACTGGGACAAGgGCCATTGTGAGAACcCTGCCTTCTGGCCACATT
Sus_scrofa_(pig)                     AAGAAATCgGTGTATACcGGGACcAGgGCCATTGTGAGAACTCTGCCTTCTGGtCACATT
Homo_sapiens_(human)                 AAGAAATCTGTGTATACTGGaACAAGAGCaATTGTGAGAACTCTGCCTTCTGGCCACATT
Macaca_mulatta_(Rhesus_monkey)       AAGAAATCTGTGTATACcGGcACAAGAGCaATTGTGAGgACTCTGCCTTCTGGCCACATT

Mus_musculus_(house_mouse)           GGGCcaGCTGCTTGGAaTTACgTtGATCAGAAGAaAgcTGGTCTCcTAtgGCCTTGTGGG
Rattus_norvegicus_(Norway_rat)       GGGCTGGCTGCTTGGAGTTACgTcGATCAGAAGAaAgcTGGTCTCaTgtgGCCTTGTGGG
Sus_scrofa_(pig)                     GGGCTGGgaGCTTGGAGTTACATtGATCAGAAGAGAAATGGTCTCTcACTGCCTTtcaGG
Homo_sapiens_(human)                 GGGCTGaCTGCaTGGAGTTACATAGATCAGAAGAGAAATGGTCcCTTACTGCCTTGTGGG
Macaca_mulatta_(Rhesus_monkey)       GGGCTGatTGCaTGGAGTTACATAGATCAGAAGAGAAATGGTCcCTTACTGCCTTGTGGG

Mus_musculus_(house_mouse)           AatGTAATGGGAaCtCTGTCAgCAaTGGAcATAAGGCAAaGTGGGAGCCAACGTCTGTCt
Rattus_norvegicus_(Norway_rat)       AatGgAATGaGACCtCTGTCcACAGTGGAcgTAAGGCAAaGTGGGAGaCAgCGTCTGTCt
Sus_scrofa_(pig)                     AGAGTcATGGGACggCTGTCAACAGTGGtGATcAGGCAAGaTGGGcGCCAgtGTCTGTCA
Homo_sapiens_(human)                 AGAGTAATGGaACCcCcGTCAACAGTGGAGATAAGGCAAGaTGGGAGCCAACGTCTGTCA
Macaca_mulatta_(Rhesus_monkey)       AGAGTAATGGaACCcCTaTCAACAGTGGAGATAAaGCAAGGTGGGAGCCgACGTCTGTCA

Mus_musculus_(house_mouse)           GAAGCCCAGTGGTgcCtg
Rattus_norvegicus_(Norway_rat)       GAAGCCCAGTGGTgTCt-
Sus_scrofa_(pig)                     GAAGCCCAcTGGTATCC-
Homo_sapiens_(human)                 GAAGCCCAGTGGTATCCT
Macaca_mulatta_(Rhesus_monkey)       GAAGCCCAGTGGTATCCT
```

Table S3 List of proteins of mass spectrometry results (score>100)

Num	Prot_acc	Prot_desc	Prot_score
1	P07900	Heat shock protein HSP 90-alpha	153
2	Q6S8J3	POTE ankyrin domain family member E	131
3	Q9P212	1-phosphatidylinositol 4,5-bisphosphate phosphodiesterase epsilon-1	117
4	P23396	40S ribosomal protein S3	115
5	P68371	Tubulin beta-4B chain	114
6	P02545	Prelamin-A/C	110
7	P07437	Tubulin beta chain	106
8	P62851	40S ribosomal protein S25	103

Table S4 The primers for qRT-PCR

Gene	Forward primer	Reverse primer
18S	CGGCTACCACATCCAAGGAA	GCTGGAATTACCGCGGCT
circPICALM1	GGTGATATACCAGACCTTTCACAGA	TGCCAACTGTGGGATGTTCA
circFAM208A	TGCAGCCTTTATGAAGTTGTGG	ATTCTCGAGAGCCTGGAGTT
circ PLCE1	CCTTACTGCCTTGTGGGAGA	GGGATTGCACTCTGCAACTT
circ EPT1	GGAGTTGAGGCCTGGTATGA	CGCCAGCCAAGTAGGAAATA
circPICALM2	GCCCAATGATCTGCTTGATT	TTGAAGACCACCACCCAACT
circ UBA2	TGAAAGTGGAACAGCTGGGT	GCAACCTGTGCCTTTGATCT
PLCE1	CTGCCAAGACCTTTTTGAGCC	CCATGCAGTCAGCCCAATGT
hHPRT	TTCCTTGGTCAGGCAGTATAATCC	AGTCTGGCTTATATCCAACACTTCG

Table S5 The primers for vector construction

Name	Forward primer	Reverse primer
circPLCE1	CGGAATTCTAATACTTTCAGGAAACAGGAATCATTCAAACTGGATTTTAA	CGGGATCCAGTTGTTCTTACAGGATACCACTGGGCTTCTGACAGACGTTG
shcircPLCE1-1	CCGGGTATCCTGAAACAGGAATCATCTCGAGATGATTCCTGTTTCAGGATACTTTTTG	AATTCAAAAAGTATCCTGAAACAGGAATCATCTCGAGATGATTCCTGTTTCAGGATAC
shcircPLCE1-2	CCGGGCCCAGTGGTATCCTGAAACACTCGAGTGTTTCAGGATACCACTGGGCTTTTTG	AATTCAAAAAGCCCAGTGGTATCCTGAAACACTCGAGTGTTTCAGGATACCACTGGGC
circPLCE1-Flag	CGGAATTCTAATACTTTCAGGACGACGATAAGTAAAATCATTGCAAATCAGTGATGG	CGGGATCCAGTTGTTCTTACATCCTTGTAATCAAATCCAGTTTGAATGATTCCTGTTTCAGGATACCACTGGGC
circPLCE-411aa-Flag	CCCGGACGAATTCTTCGAAATGACTTCTGAAGAAATGACAGCTTCTGTT	TGCGGATCACTAGTGCTAGCTTACTTATCGTCGTCATCCTTGTAATCAAATCCAGTTTGAATGATTCCTGTTTCAGGATACCA
IRES	AAAAATGAACAATGACTCGAGAATCATTGCAAATCAGTGATGGAT	TTTCATTGCCATACGGAATTCCTTGGACTAACACACAGGTTTTGA
IRES-del-1	AAAAATGAACAATGACTCGAGAATCATTGCAAATCAGTGATGGAT	TTTCATTGCCATACGGAATTCATTTTCTTAGTTATAACTTCCTAT
IRES-del-2	AAAAATGAACAATGACTCGAGTTATTTGCCTCTTAATGCTCCTGA	TTTCATTGCCATACGGAATTCCTTGGACTAACACACAGGTTTTGA
HA-HSP90α (FL)	GGGAGACCCAAGCTGGCTAGCATGCCCCCGTGTTCGGCGGGGACGGC	TAGTCCAGTGTGGTGGAATTCTTAAGCGTAGTCTGGGACGTCGTATGGGTAGTCTACTTCTTCCATGCGTGATGT
HA-HSP90α (N)	GGGAGACCCAAGCTGGCTAGCATGCCCCCGTGTTCGGCGGGGACGGC	TAGTCCAGTGTGGTGGAATTCAGCGTAGTCTGGGACGTCGTATGGGTATTTCAGGTGTAGGATAACTTTTGTTCC
HA-HSP90α (C)	GGGAGACCCAAGCTGGCTAGCGAGTACTTGGAGGAACGAAGAATAAAG	TAGTCCAGTGTGGTGGAATTCAGCGTAGTCTGGGACGTCGTATGGGTAATCTCCTTCAAGGGGTGGCATTTCTTC
His-RPS3	GGGAGACCCAAGCTGGCTAGCATGGCAGTGCAAATATCCAAGAAGAGG	TAGTCCAGTGTGGTGGAATTCTTAATGGTGATGGTGATGATGTGCTGTGGGGACTGGCTGGGGCAT
Myc-HSP70	GGGAGACCCAAGCTGGCTAGCGAGATCTTCTCCGCCCCCGCTACCGGC	TAGTCCAGTGTGGTGGAATTCTTCCTTATCGTCGTCATCCTTGTAATCAAGTTTAAGACAGGTTTATTTTAT

Table S6 The probe sequence for circPLCE1 FISH and ISH

Gene	**Sequence**
circPLCE1	GTTTGAATGATTCCTGTTTCAGGATACCACTGGGCTTCTG

Supplemental Methods

RNA extraction and real-time PCR

Total RNA was isolated from cells by TRIzol Reagent (Thermo Fisher Scientific). ReverTra Ace qPCR RT Kit (Toyobo) was used to perform reverse transcription according to the manufacturer's instructions. The nuclear and cytoplasmic fractions were isolated by NE-PER™ Nuclear and Cytoplasmic Extraction Reagents (Thermo Scientific).The Applied Biosystems 7500 Sequence Detection system was used to carry out quantitative real-time reverse transcription PCR (qRT-PCR) with the SYBR Green PCR Master Mix (Applied Biosystems). We generated standard curves and applied the $2^{-\triangle\triangle CT}$ method with normalized to 18S rRNA. We next used the gene of human hypoxanthine-guanine-phosphoribosyltransferase (hHPRT) to quantify cancer metastasis in mouse livers. All the gene-specific primers were obtained from Invitrogen and the oligonucleotide sequences are listed in Table S4.

RNase R treatment

RNase R (Epicentre Technologies, Madison, WI, USA) was used to assess the stability of circRNA. Total RNA (2 μg) was mixed with 0.6ul 10 × RNase R Reaction Buffer and 0.2 μl RNase R or DEPC-treated water (control group). The samples were then incubated at 37 ℃ for 15 min1. The expression levels of circPLCE1 and linear PLCE1 were detected by qRT-PCR.

Actinomycin D assay

For the half-life of circRNA assessment, the gene transcription was blocked by adding 2mg/mL Actinomycin D (Sigma-Aldrich, St. Louis, MO, USA) to the cell culture medium. DMSO was used as a negative control. Cells were harvested at 0, 4, 8, 12, 24h and the stability of circPLCE1 and linear PLCE1 was analyzed by qRT-PCR.

RNA fluorescence in situ hybridization (FISH) assay

According to the manufacturer's instructions, the FISH kit (Ribo Bio, Guangzhou, China) was utilized to perform FISH in cells and the results are visualized by confocal

microscopy laser-scanning microscope (Leica TCS-SP8, Leica Microsystems Inc, Buffalo Grove, IL, USA). CRC cells were fixed with 4% paraformaldehyde at room temperature for 10 min, treated with 0.5% Triton X-100 in phosphate buffer saline (PBS) at 4 ℃ for 5 min and then pre-hybridized by Pre-hybridization Buffer at 37 ℃ for 30 min. Finally the cells were hybridized with 2.5 ul 20uM circPLCE1 FISH probes (GenePharma, Shanghai, China) overnight at 37 ℃. The probe sequences are shown in Table S6.

RNA in situ hybridization (ISH) assay

According to the manufacturer's instructions, the ISH Detector kit (BSTER, Wuhan, China) was used to perform ISH in paraffin-embedded CRC tissues. CRC tissues were fixed with 4% paraformaldehyde at room temperature for 10 min; then they were digested with proteinase K at 37 ℃ for 2 min and pre-hybridized at 37 ℃ for 3 h. The tissues were hybridized with double 5'-3'-digoxin (DIG)-labeled circPLCE1 ISH probes (TSINGKE Biological Technology) overnight at 37 ℃. Finally, tissues were incubated with an anti-digoxin monoclonal antibody conjugated with alkaline phosphatase and then incubated with 3, 3'-diaminobenzidine (DAB).

Plasmids construction, transfection and lentivirus infection

For RPS3, HSP70 and HSP90α-expressing plasmids, the full-length ORF sequences with tag of these three genes were respectively subcloned into the pcDNA3.1 vector. The activity of the internal ribosome entry site (IRES) was detected with pCMV-IRES-Renilla Luciferase-IRES-Gateway-Firefly Luciferase (pIRIGF) vector. CRC cells were transfected with above vectors using Lipofectamine® 3000 (Invitrogen) according to the manufacturer's instructions. To generate the circPLCE1 overexpression plasmid, the full-length circPLCE1 cDNA was cloned into pLO-ciR vector (Geneseed Biotech, Guangzhou, China). For construction of shcircPLCE1 plasmids, shcircPLCE1 sequences were cloned into pLKO.1 vector. All constructs were verified by sequencing. For stable lentivirus infection, HEK293T cells were transfected with the indicated plasmids, psPAX2 and pMD2G (Addgene) according to

the manufacturer's instructions. Then CRC cells were infected with above supernatant in the presence of polybrene and selected with puromycin after infection. The oligonucleotide sequences for vector construction are listed in Table S5.

Cell proliferation assays

Cell proliferation was examined using plate colony formation, 3D anchorage-free colony formation, soft agar cell colony formation and patient-derived organoids (PDOs) growth. For plate colony formation, 500 cells were plated into 6-well platesand cultured for two weeks. Then the colonies were fixed with methanol and stained with 0.1% crystal violet for 15 minutes at room temperature. Cell colonies were counted and imaged. For 3D anchorage-free colony formation assay, 5,000 cells were seeded in 200 μL DMEM supplemented with 10% FBS in 96-well ultra-low attachment microplate (7007; Corning, USA). Cell culture media was refreshed every three days, and images of 3D colonies were captured using a phase-contrast microscope (DMI4000B, Leica, Wetzlar, Germany). The volume of 3D colonies was calculated using the formula: Volume = 4/3πR3. For soft agar cell colony formation assay, 5,000 cells were suspended in 75 μL 0.4% agar DMEM solution and seeded over 50 μL of bottom agar in 96-well plates. Images of colonies were captured after 10 days using a phase-contrast microscope.

Migration and invasion assays

Cell migration and invasion were examined using cell migration assays and wound healing assay. Cell migration assays were performed with 24-well plates with 8-μm pore size chamber inserts (Corning). In general, 5×10^4 cells resuspend with 200 μl serum-free DMEM were seeded in the upper chamber well and 800 μl of DMEM with 10% FBS was added into the lower chamber. After 24 h, cells migrating through the membrane were fixed with 4% paraformaldehyde for 15 min, and then stained with 0.1% crystal violet for 15 min. The cells were viewed under an inverted microscope (DMI4000B, Leica, Wetzlar, Germany) and quantified using software ImageJ. For wound healing assay, a total of 2×10^6 cells were seeded in six-well plates and

incubated until confluency was reached. A 100ul pipette tip was used to create a rectilinear scratch. After 12 h, cells were fixed with 4% paraformaldehyde for 15 min, and then stained with 0.1% crystal violet for 15 min. An inverted microscope (DMI4000B, Leica, Wetzlar, Germany) was utilized to image the wound closure.

Immunohistochemistry (IHC)

Paraffin-embedded tissues were deparaffinized with dimethylbenzene followed by antigen retrieval. The tissues were blocked with normal goat serum at 37℃ for 30 min. Next, the tissues were incubated overnight at 4°C with specific primary antibodies against p-P65 (ab194726, 1:100), RPS3 (ab128995, 1:100), and ki67 (ab16667, 1:200). Finally, the tissues were incubated with appropriate secondary antibodies and then incubated with 3, 3'-diaminobenzidine (DAB).

Western blot

Cell and tissue samples were lysed with radio-immunoprecipitation assay buffer (RIPA) with protease and phosphatase inhibitors cocktail (Promega). Proteins were separated by SDS-PAGE and then transferred to polyvinylidene fluoride (PVDF) membranes by the Trans-Blot System (Bio-Rad, CA, USA). The membranes were blocked by milk and then incubated with specific primary antibodies against PLCE1 (Sigma#HPA015597), Flag (CST#14793S, 1:1000), HSP90α (CST#4877, 1:1000), RPS3 (CST#9538, 1:1000), GAPDH (CST#5174, 1:1000), Ubiqitin (CST#58395, 1:1000), HA (CST#3724, 1:1000), Myc (CST#2276, 1:1000), His (CST##12698, 1:1000) and P65 (CST#8242, 1:1000). Finally membranes were incubated with a specific secondary antibody and visualized by ECL Blotting Detection Reagents. GAPDH served as a control for western blot analysis.

Immunoprecipitation assay

For immunoprecipitation, the cells transfected with indicated plasmids were lysed in Pierce IP lysis buffer containing protease inhibitors cocktail (Thermo Scientific, IL, USA) for 30 min at 4℃. Cell lysates were centrifuged at 13,000g for 20 min at 4℃.

Protein A/G Magnetic Beads (Thermo Scientific) were prewashed with washing buffer two times. Then, indicated antibodies were added to the beads for antibody crosslink 4h at 4 ℃. The cell lysates were added to the antibody-crosslinked beads overnight at 4 ℃. After washing with washing buffer for five times, the beads were incubated with 2x sample loading buffer and boiled for 10 minutes. Finally, the lysate samples were resolved on SDS-PAGE for further study.

Mass spectrometry assay

The gel was chopped into small fragments with a razor blade, destained, and subjected to digestion by modified porcine trypsin (50–100 ng/digestion; Promega). After trypsin digestion, peptides were dissolved in 0.1% FA and 2% ACN, directly loaded onto a reversed-phase analytical column (75 um i.d. x 150mm, packed with Acclaim PepMap RSLC C18, 2 um, 100Å, nanoViper). The gradient was comprised of an increase from 5% to 50% solvent B (0.1% FA in 80% ACN) over 40 min, and climbing to 90% in 5 min, then holding at 90% for the 5 min. All at a constant flow rate of 300 nl/min. The MS analysis was performed on Q Exactive hybrid quadrupole-Orbitrap mass spectrometer (Thermo Fisher Scientific). The peptides were subjected to NSI source followed by tandem mass spectrometry (MS/MS) in Q ExactiveTM (Thermo) coupled online to the UPLC. Intact peptides were detected in the Orbitrap at a resolution of 70,000. Peptides were selected for MS/MS using NCE setting as 27; ion fragments were detected in the Orbitrap at a resolution of 17,500. A data-dependent procedure that alternated between one MS scan followed by 20 MS/MS scans was applied for the top 20 precursor ions above a threshold ion count of 1E4 in the MS survey scan with 30.0s dynamic exclusion. The electrospray voltage applied was 2.0 kV. Automatic gain control (AGC) was used to prevent overfilling of the ion trap; 1E5 ions were accumulated for generation of MS/MS spectra. For MS scans, the m/z scan range was 350 to 1800 m/z. Fixed first mass was set as 100 m/z. Protein identification were performed with MASCOT software by searching Uniprot_Aedis Aegypti.
